# Wild edible plant species and their role in nutrition and health in Korahe Zone, Eastern Ethiopia

**DOI:** 10.1186/s41182-025-00867-6

**Published:** 2025-12-05

**Authors:** Getu Alemayehu, Ashebir Awoke, Zewdie Kassa

**Affiliations:** 1 Department of Biology, Jigjiga University, P.O. BOX 1020, Jigjiga, Ethiopia; 2 Department of Biology, Mizan-Tepi University, P.O. Box 121, Tepi, Ethiopia; 3 Department of Biology, Salale University, P.O. Box 245, Fitche, Ethiopia

**Keywords:** Conservation, Ethiopia, Ethnobotany, Food security, Korahe zone, Somali communities, Wild edible plants

## Abstract

**Background:**

Wild edible plants (WEPs) are vital for food security, nutrition, and cultural identity, particularly in arid and semi-arid regions. In Korahe Zone, Eastern Ethiopia, Somali communities rely heavily on natural vegetation, yet ethnobotanical documentation of WEPs remains limited. This study aimed to investigate the diversity, utilization, knowledge patterns, and conservation status of WEPs, with implications for food security and health.

**Methods:**

Ethnobotanical data were collected from 120 purposively selected informants across five study sites using semi-structured interviews, focus group discussions, and guided field walks. Species were also screened against the IUCN Red List to identify threatened and vulnerable wild edible plants, providing insights into their conservation status and informing sustainable management strategies. Quantitative indices, including the Botanical Ethnoknowledge Index (BEI), Relative Frequency of Citation (RFC), Informant Consensus Factor (ICF), and Jaccard Similarity Index (JSI), were applied. Preference ranking, direct matrix ranking, and priority ranking were used to assess species use, multifunctionality, and perceived threats. Statistical analyses (*t* tests, ANOVA, and Pearson correlation) were conducted to examine variations in ethnobotanical knowledge across gender, age, literacy, and experience.

**Results:**

A total of 57 WEP species across 22 families were documented, with shrubs and trees dominating. Fruits were the most commonly consumed part, while roots, tubers, leaves, stems, and resins were used during food scarcity. Knowledge varied significantly by age, gender, literacy, and experience, with older, male, and key informants exhibiting greater familiarity. *Cordeauxia edulis* Hemsl., *Balanites aegyptiaca* (L.) Delile, *Amaranthus dubius* Mart. ex Thell., and *Moringa stenopetala* (Baker f.) Cufod*.* were highly preferred and multipurpose. Overharvesting, habitat loss, climate variability, grazing, and invasive species were major threats. Three species were identified as threatened either critically endangered or vulnerable, highlighting the urgent need for their conservation and sustainable management.

**Conclusions:**

WEPs are crucial for local nutrition, food security, and cultural heritage. Integrating indigenous knowledge with *in-situ* and *ex-situ* conservation strategies and promoting sustainable use and cultivation of high-value species is essential to safeguard these resources for future generations.

**Supplementary Information:**

The online version contains supplementary material available at 10.1186/s41182-025-00867-6.

## Background

Plants provide essential livelihoods and food for nearly 300 million people worldwide through non-timber forest products (NTFPs) [1–4]. In tropical and low-income regions, NTFPs, including wild edible plants (WEPs), are crucial sources of food, medicine, and income [5–10]. WEPs sustain rural households during food shortages, reduce vulnerability to environmental stresses, alleviate nutrient deficiencies, diversify diets, and many possess medicinal properties, earning them the designation “nutraceutical plants” [11, 12].

For centuries, WEPs have supported local food systems and cultural traditions, contributing to food security and poverty reduction [13–16]. However, knowledge of their use is rapidly eroding due to land-use change, urbanization, industrialization, and rural outmigration [17, 18]. In the Korahe Zone, cultural beliefs and taboos further limit domestication and wider use of WEPs. Some species are considered “famine foods” and avoided due to associated poverty or social stigma, while others are believed to belong “to the wild” and should not be cultivated near homes. Certain fruits are thought to be gender-specific for collection, and some species are avoided, because they are believed to host protective spirits or attract wildlife [19, 20].

Despite their importance, WEPs remain poorly documented and undervalued in national statistics and policy frameworks [3, 21]. Neglecting these resources reduces opportunities to alleviate poverty, ensure food security, diversify agriculture, and sustain rural livelihoods. Incorporating WEPs into agroforestry systems and home gardens, particularly in drylands, can enhance food security while conserving biodiversity. Systematic ethnobotanical documentation is, therefore, essential to preserve indigenous knowledge and promote sustainable use.

In Ethiopia, food insecurity persists due to recurrent droughts, poverty, and fragile socio-economic conditions. Although WEPs are widely used, they are largely excluded from food and nutrition policies. A systematic review identified 651 WEP species in Ethiopia, with fruits as the most common edible part [22]. Overgrazing, land conversion, and declining cultural practices threaten their survival. WEPs provide essential protein, vitamins, and health-promoting compounds during droughts and famines but are often stigmatized as “food for the poor” [23–25]. Lulekal et al. documented 413 WEP species, noting that fewer than 5% of Ethiopian districts have been ethnobotanically studied, leaving major knowledge gaps, particularly in the Somali Region [9]. Case studies from Mieso and Raya-Azebo confirm their nutritional, cultural, and economic importance, yet local knowledge remains vulnerable to ecological degradation and changing land use [24, 25].

Korahe Zone is predominantly inhabited by Somali pastoral communities whose livelihoods, cultural values, and food systems are closely tied to the dryland environment [26–28]. The Somali ethnic group accounts for > 99% of the population, with Somali universally spoken. Clan-based social structures govern resource sharing, mobility, and environmental management. Pastoralism and agro-pastoralism are the main subsistence strategies for nearly 80% of residents, shaping local knowledge of WEP use and management [28]. The population is relatively young, with high dependency ratios, low school enrollment, and limited access to services, such as electricity, roads, and formal healthcare [26]. These characteristics make Korahe an ideal context for studying WEPs, as traditional ecological knowledge is integral to survival, dietary practices, and resilience to recurrent drought and food insecurity.

Preliminary nutritional analyses of selected species suggest high potential, but systematic ethnobotanical documentation is lacking. Research is urgently needed to capture species diversity, traditional uses, nutritional value, and threats to WEPs in this zone, information critical for strengthening resilience strategies and integrating WEPs into regional food security planning.

This study aligns with the United Nations Sustainable Development Goals (SDGs), particularly Goal 2 (Zero Hunger), Goal 3 (Good Health and Well-being), and Goal 15 (Life on Land). By linking biodiversity conservation and traditional ecological knowledge with food security and health, the research seeks to generate evidence that informs both policy and community resilience. Specifically, the study aims to: (i) compile a comprehensive inventory of WEPs in Korahe Zone, including vernacular names, edible parts, preparation methods, marketability, and seasonal availability; (ii) assess their cultural significance using ethnobotanical indices; (iii) examine socio-demographic variations in knowledge and use across gender, age, and livelihood systems; and (iv) identify ecological and anthropogenic threats while proposing conservation-oriented strategies aligned with the SDGs.

These objectives are guided by four hypotheses: (H₁) Korahe Zone hosts a diverse assemblage of WEPs actively integrated into local diets and cultural practices; (H₂) high-use species provide substantial nutritional contributions, particularly during lean seasons; (H₃) traditional knowledge varies significantly across socio-demographic groups, with older adults and women retaining greater expertise; and (H₄) key WEPs face mounting pressures from environmental change and human exploitation, requiring urgent conservation interventions. In addressing these aims, this study fills a critical ethnobotanical gap in the Somali Region and generates insights of direct relevance to food security, biodiversity conservation, and community well-being in Ethiopia.

## Materials and methods

### Description of the study area

**Fig. 1:**
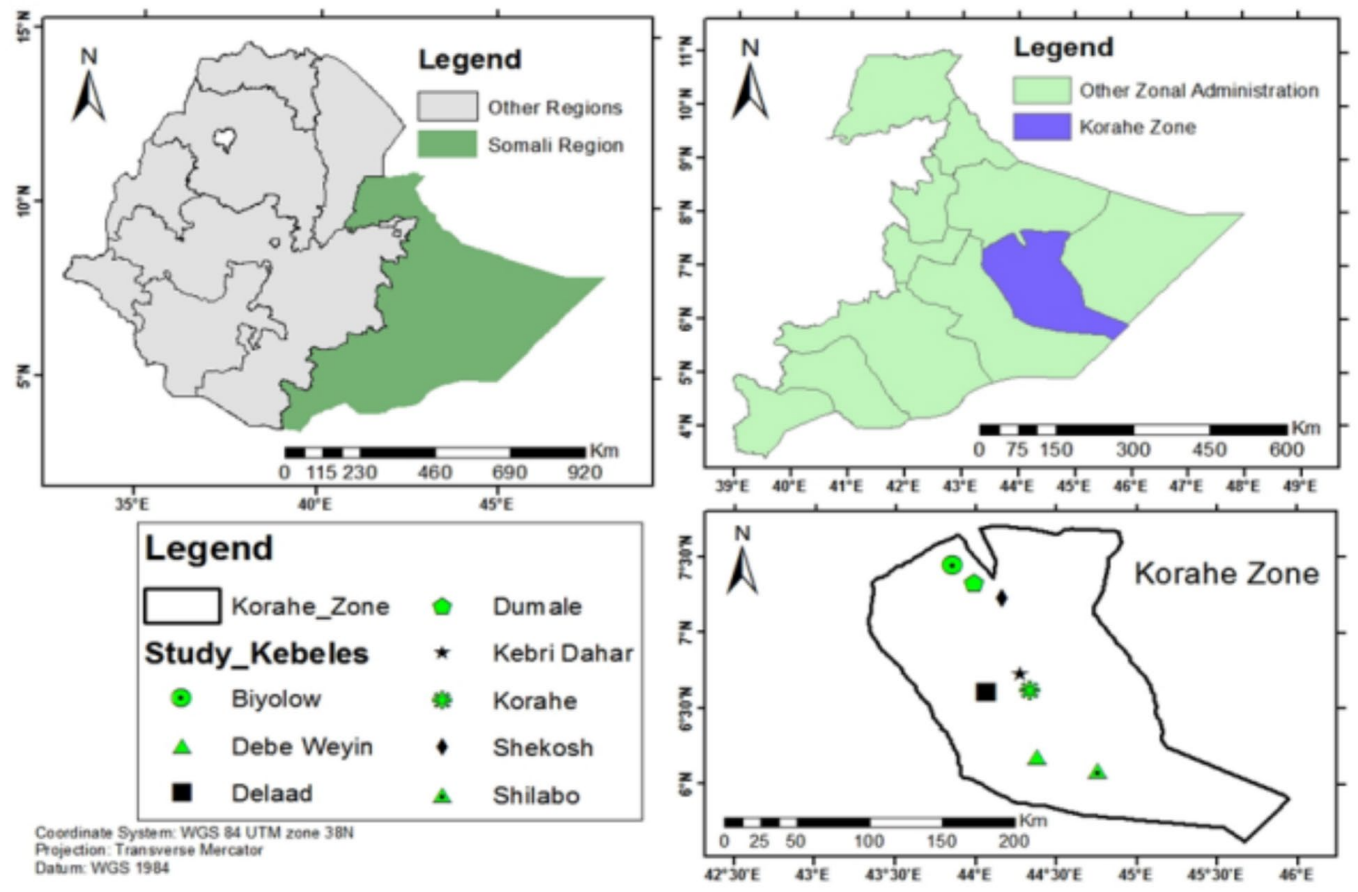
Map of the study area in Korahe Zone, Eastern Ethiopia (Source: ArcGIs 10.8)

Korahe (Somali: Qoraxeey) is one of the 11 zones of the Somali Regional State in Eastern Ethiopia. It is bordered by Shabelle Zone to the southwest, Erer Zone to the northwest, Jarar Zone to the north, Doolo Zone to the east, and the Galmudug State of Somalia to the southeast. The administrative center, Kebri Dehar, is located approximately 420 km from Jigjiga, the regional capital. Geographically, the zone lies at 6°45′N latitude and 44°30′E longitude, with elevations ranging from 489 to 493 m above sea level (Fig. 1).

Korahe Zone has a total population of 312,713, comprising 177,919 men and 134,794 women [26–28]. About 20% of the population resides in urban centers, while 80% are pastoralists. The Somali ethnic group constitutes 99.98% of the population, and Somali is the first language for nearly all residents. Islam is the predominant religion, followed by 98.92% of inhabitants.

Agro-ecologically, Korahe lies within the lowland (Qola) zone. The climate is predominantly arid to semi-arid, with an annual rainfall of 500–700 mm and mean temperatures of 27–30 °C (Korahay Meteorological Center, 2018). Rainfall is bimodal, with the Gu season (late March–May) and the Karaan season (July–September), of which the latter is generally more reliable. Rainfall is higher in the southern foothills than in the north-central plains. Strong winds, low humidity, and minimal cloud cover are characteristic features of the zone’s climate.

Topographically, the zone consists mainly of plains (46%), with mountains (40%) and rugged terrains (14%), ranging in elevation from 200 to 1500 m above sea level [26]. Sandy soils dominate (60%), followed by loamy soils (30%) [26]. Local communities classify soils based on color and fertility into four categories. A mixture of red and black soils (*Iskujirka Carra Caska iyo Carra Madowga*) is considered highly fertile and suitable for crops, such as *Guizotia abyssinica* (L.f.) Cass. and *Eragrostis tef* (Zuccagni) Trotter. Red soils (*Carra Caska*) are less fertile but still support crops, such as *Brassica carinata* A.Braun, *Mangifera indica* L., *Capsicum annuum* L., and *Zea mays* L., particularly with fertilizer application. Black soils (*Carra Madow*) are regarded as the most fertile, supporting crops such as *Allium sativum* L., *Allium cepa* L., *Zea mays L.*, and *Eragrostis tef (Zuccagni) Trotter*. Sandy soils (*Carra Cadka*), by contrast, are infertile and generally unsuitable for cultivation [27, 28].

Vegetation in Korahe is composed primarily of shrubs, woody species, and herbaceous plants, typical of lowland and midland ecosystems. Dominant vegetation types include *Acacia–Commiphora* woodlands and bushlands, alongside famine food plants, such as *Dobera glabra* (Forssk.) Juss. ex Poir., *Ziziphus mauritiana* Lam., *Grewia penicillata* Chiov., *Caralluma sprengeri* (E.Dammann and Sprenger) N.E.Br., *Sterculia rhynchocarpa* K.Schum., *Commiphora lughensis* Chiov., *Opuntia* spp., and *Delonix elata* (L.) Gamble. Local people further classify vegetation based on density, distribution, and landform association. Dense woodlands (*Dhulka Keeymaha*), once common but now scarce due to agricultural expansion, are characterized by tall trees, such as *Delonix elata* (L.) Gamble and *Ziziphus mauritiana* Lam. Open woodlands and shrublands (*Dhulka Banaanka*) are composed of scattered trees, bushes, and herbaceous species, often found near agricultural fields and escarpments, with typical plants, including *Grewia penicillata* Chiov*.*, *Z. mauritiana*, and *G. tenax*. Grasslands (*Dhulka Daaqa*) are dominated by grasses and serve primarily as grazing land [29].

## Methods of data collection and analysis

### Reconnaissance survey, study site, and informant selection

A reconnaissance survey was conducted from 15 to 28 February 2023 to gather baseline information and guide the selection of study sites. Initial data were obtained from the Korahe Zone Office of Agriculture, supplemented by consultations with local elders, kebele (administrative unit) leaders, forest guards, and other knowledgeable community members. Korahe Zone was purposively selected due to its relatively rich vegetation, high reliance on wild edible plants (WEPs), and the absence of formal conservation initiatives targeting these resources.

Based on vegetation cover and the presence of knowledgeable individuals, eight study sites were selected: Dumale, Kebri Dehar Zuria, Shekosh, Shilabo, Biyolow, Debe Weyin, Delaad, and K’orahe (Table 1).

**Table 1:** Summary of selected study sites and sociodemographic characteristics of informants

Name of sites	Altitude	GPS Coordinates		Gender		Ethnicity (Somali)	Age categories			Language (Af Soomaali)	Ocu (LH,FA,MC,CS,FW,HW)	Education level		NH	AE
		Latitude (N,S)	Longitude (E,W)	M	F		20–35	36–50	51–80						
												Illi	Lit		
Shilabo	403 m	6°05′59"N	44°46′05"E	8	7	Somali	3	5	7	Af Soomaali	LH,FA,MC,CS,FW,HW	9	6	1512	Low-land
Shekosh	884 m	7°21′58"N	43°51′22"E	9	6	Somali	2	4	9	Af Soomaali	LH,FA,MC,CS,FW,HW	10	5	2777	Low-land
K’orahe	470 m	6°33′03"N	44°22′16"E	8	7	Somali	1	6	8	Af Soomaali	LH,FA,MC,CS,FW,HW	10	5	1409	Low-land
KebreDehar zuria	534 m	6°44′07"N	44°15′30"E	9	6	Somali	3	4	8	Af Soomaali	LH,FA,MC,CS,FW,HW	9	6	3015	Low-land
Dumale	375 m	5°58′51"N	44°25′34"E	8	7	Somali	3	5	7	Af Soomaali	LH,FA,MC,CS,FW,HW	10	5	1272	Low-land
Dalad	561 m	6°40′10"N	44°36′27"E	9	6	Somali	2	4	9	Af Soomaali	LH,FA,MC,CS,FW,HW	9	6	1432	Low-land
Debe Woyin	408 m	6°09′22"N	44°23′14"E	9	6	Somali	1	6	8	Af Soomaali	LH,FA,MC,CS,FW,HW	10	5	1368	Low-land
Biyolow	848 m	6°57′57"N	43°47′27"E	8	7	Somali	3	4	8	Af Soomaali	LH,FA,MC,CS,FW,HW	9	6	1510	Low-land
Total				68	52		18	38	64			76	44	14,295	

*Illi* = illiterate, *Lit* = literate, *M* = Male, *F* = Female, *AE* = Agro-ecology, *NH* = Number of households, *Ocu* = Occupation *LH* = livestock holders, *FA* = farmers, *MC* = merchants, *CS* = Charcoal sellers, *FW* = fire wood sellers, *HW* = House wife

A total of 120 informants, aged 20–80 years, participated in the study. Fifteen participants were selected from each kebele. Among them, 88 were general informants identified through snowball sampling, prioritizing long-term residents with indigenous knowledge of WEPs [18, 30]. An additional 32 key informants were purposively selected based on recommendations from elders, local administrators, agricultural officers, and development experts. Key informants included traditional gatherers, vendors, cooks, elders, and other community members with specialized knowledge and experience in the use and management of WEPs.

### Ethnobotanical data collection and plant identification

Ethnobotanical data were collected during three field visits to eight study sites between May 2023 and April 2024 using semi-structured interviews, guided field walks, focus group discussions (FGDs), and market surveys. A semi-structured checklist prepared in English and translated into Somali guided interviews (Fig. S1) and included: (a) personal information (name, age, gender, education, and address) and (b) ethnobotanical knowledge (vernacular names, plant parts used, and conditions of use). Interviews were conducted in Somali with the support of four native-speaking translators at times and locations chosen by informants. Both individual and group interviews explored uses of wild edible plants (WEPs), perceived threats, and local management practices. Permission for plant collection and data gathering was obtained from landowners and district administrative and agricultural offices following ethical guidelines.

Voucher specimens were collected with the assistance of informants and deposited at the Mini Herbarium of Jigjiga University. Specimens were pressed, air-dried, labeled, and processed using heater–drying and deep-freezing techniques. Preliminary identification was done in the field to family or species level, with further verification at the National Herbarium (ETH), Addis Ababa University, using the Flora of Ethiopia and Eritrea, authenticated herbarium specimens, and standard taxonomic keys [31, 32]. Botanical names were cross-checked using the International Plant Names Index (IPNI), Plants of the World Online (POWO), and additional digital resources, including PlantNet, Flora Finder, PlantSnap, Google Image Search, the African Plant Database, the World Checklist of Selected Plant Families, and JSTOR Global Plants [18, 30, 33].

Four FGDs, each comprising 6–8 participants aged 20–75 years of mixed gender, were conducted with key informants recognized for their ethnobotanical knowledge. Open discussions documented plant species consumed as food, vernacular names, edible parts, preparation methods, availability, and local management and conservation practices (Fig. S2).

Guided field walks, or “walk-in-the-woods” activities, were conducted with informants to locate and identify WEPs in their natural habitats. Voucher specimens were collected, labeled with local names, and processed for verification. Identification was based on morphological features (leaf shape, flowers, fruits) using standard regional floras, with assistance from local experts and community elders. Data recorded included vernacular names, edible parts, preparation methods, growth habits, and ecological settings, supported by photographs (Fig. S3). Discussions also addressed threats to WEPs, conservation practices, and intergenerational knowledge transfer.

To complement field data, a market survey was conducted in three local marketplaces. Semi-structured interviews with vendors captured diversity, availability, quantities, sources, pricing, patterns of use, and market demand of WEPs. The survey provided insights into the commercial value of WEPs, their role in household economies, and challenges related to conservation and sustainability (Fig. S4).

### Quantitative ethnobotanical analysis

To complement qualitative data, several quantitative ethnobotanical tools were employed to evaluate the cultural importance, knowledge distribution, and conservation status of wild edible plants (WEPs) in the study area.

### Botanical ethnoknowledge index (BEI)

The Botanical Ethnoknowledge Index (BEI) was employed to quantify the extent and distribution of ethnobotanical knowledge within the study population and to allow comparisons across groups inhabiting similar ecological and floristic settings [34]. The BEI integrates multiple variables, including: (i) the total number of plant species reported by all members of a group, (ii) the mean number of species cited per participant, (iii) the mean number of citations per species, (iv) the number of participants in each group, and (v) the total number of species reported across all groups in the study.

The index produces values ranging from just above 0 to a theoretical maximum of 2. Higher BEI values reflect a greater depth and breadth of ethnobotanical knowledge within the group. While values approaching 2 are possible, they are rare and generally indicate distinct or highly specialized knowledge relative to other groups.

The BEI values for the study sites were calculated using the following equation:

1 $${\text{BEI}}\,{ = }\left( {\frac{{{\text{ms}}}}{{{\text{sg}}}}\,{ + }\,\frac{{{\text{ms}}}}{{\text{N}}}} \right)\, \times \,\frac{{{\text{sg}}}}{{{\text{st}}}}$$

In Eq. 1, BEI refers to the Botanical Ethnoknowledge Index. ms denotes the mean number of species reported per participant within a particular group, while Sg represents the total number of species reported by all participants in that group. mc is the mean number of citations per species, and N is the total number of participants in the group. St indicates the total number of species reported across all groups included in the study.

### Jaccard similarity index (JSI)

The Jaccard Similarity Index (JSI) was applied to compare the composition of wild edible plants (WEPs) documented in this study with those reported from other regions of Ethiopia. This index measures the proportion of shared species between two areas relative to the total number of species recorded, thereby providing insights into floristic overlap and regional variation in WEP use. The JSI was calculated using the following equation:

2 $${\text{JSI}} = \frac{{\text{c}}}{{{\text{a}} + {\text{b}} - {\text{c}}}} \times 100$$

In Eq. 2, JSI represents the Jaccard Similarity Index, which measures the degree of similarity between two study areas. In this formula, a is the number of plant species recorded in the current study area, b is the number of plant species documented in the comparison study area, and c is the number of species shared by both areas. The JSI ranges from 0 to 1, where a value of 1 indicates complete similarity and 0 indicates no similarity between the two sites. For ease of interpretation, the JSI can be multiplied by 100 to express the similarity as a percentage, providing a clearer representation of similarity levels [35, 36].

### Informant consensus factor (ICF)

The informant consensus factor (ICF) was calculated to assess the level of agreement among informants on the use of wild edible plants (WEPs) across different use categories. ICF values above 0.7 indicate strong consensus and widely shared traditional knowledge, while values below 0.5 reflect more diverse or less consistent knowledge among participants [37]. The ICF was computed using the following equation:

3 $${\text{ICF }} = \frac{{{\text{Nur}} - {\text{Nt}}}}{{{\text{Nur}} - 1}}$$

In Eq. 3, Nur refers to the number of use reports from informants for a particular use category, and Nt refers to the number of taxa that are used for a particular use category by all informants. The calculated values of ICF always lie between 0 and 1; the values close to 1 mean that there is high agreement of informants in WEPs, and low ICF values indicate that informants disagree over the use of WEPs.

### Relative frequency of citation (RFC)

The Relative Frequency of Citation (RFC) was used to assess the local importance of each species based on how frequently it was mentioned by informants. RFC values range from 0 (no mention) to 1 (maximum mention), with higher values reflecting greater cultural salience and reliance on a particular species [37]. The RFC was calculated using the following equation:

4 $${\text{RFC}} = \frac{{{\text{FC}}}}{{\text{N}}}$$

In Eq. 4, FC = the number of informants citing the use of the species and N = the total number of respondents participating in the survey.

### Preference ranking

Preference ranking of wild edible plants (WEPs) was conducted following established ethnobotanical methods [38, 39]. Key informants were asked to evaluate the most frequently cited WEPs based on taste, availability, accessibility, cultural significance, and income-generating potential. Each informant assigned scores ranging from 1 (least preferred) to 10 (most preferred). The species considered having the sweetest taste or highest desirability received a score of 10, while the least preferred species received a score of 1. Scores from all informants were summed to determine the overall rank of each species. This method provided insight into the culturally and nutritionally most valued WEPs in the study communities.

### Direct matrix ranking (DMR)

In addition to their dietary role, many WEPs serve multiple functions in local households. To assess these diverse contributions, direct matrix ranking was applied to six multipurpose species, following [38, 39]. Key informants assigned scores from 0 (no use) to 5 (highest use) across five categories: food, building, medicine, firewood, and charcoal. The average values for each category were calculated, and cumulative scores were used to rank the species according to their multifunctional importance. This approach highlighted the species most relied upon by local communities and identified those under the greatest utilization pressure.

### Priority ranking of threats

To identify the main threats affecting WEPs, priority ranking was conducted with key informants. Six major threats were considered: agricultural expansion, charcoal production, introduction of exotic species, firewood collection, overgrazing, and persistent drought. Informants scored each threat on a scale of 1 (least severe) to 6 (most severe), following standard ethnobotanical procedures [38, 39]. Scores were summed across informants to produce a composite ranking, which revealed the most critical drivers of WEP decline. This method provided a clearer understanding of the ecological, economic, and cultural pressures influencing both the utilization and conservation of wild edible plants in the study area.

### Data analysis

Field data were systematically organized and categorized, including the local and scientific names of plants, their botanical families, life forms, edible parts, and habitats. All records were maintained using Microsoft Word 2019. Descriptive statistics were applied to summarize the data, and frequency distributions were presented using tables, pie charts, and bar graphs. Statistical analyses were performed in R software (version 4.4.2).

To assess variation in ethnobotanical knowledge across the different study sites, the Botanical Ethnoknowledge Index (BEI) was applied. Normality of the data was assessed using the Shapiro–Wilk test prior to parametric analyses. Independent *t* tests were conducted to evaluate differences in knowledge of wild edible plants between genders, across educational levels, and between participants with and without wild edible plants. One-way ANOVA was used to examine knowledge variation among different age groups, while Pearson’s correlation analysis assessed the relationship between age and the number of wild edible plants reported [18].

### Ethical considerations

Prior to fieldwork, official permission was obtained through a support letter from the Department of Biology, Jigjiga University, and approved by the administrative offices of Korahe Zone. The study was conducted in full compliance with the Code of Ethics of the International Society of Ethnobiology (ISE). Prior informed consent was obtained from all participants after clearly explaining the study’s objectives, scope, and significance. Participants were assured that their involvement was voluntary and that the research had no commercial intent, being undertaken solely for academic and community benefit.

Cultural values and traditional knowledge systems were respected throughout the study. Informants were informed that their contributions would support the documentation and preservation of ethnobotanical knowledge on wild edible plants, which are essential for local food security, cultural identity, and biodiversity conservation. Emphasis was placed on promoting the sustainable use of these resources for the well-being of both current and future generations.

## Results

### Sociodemographic characteristics of informants

Informants were grouped into three age categories: young adults (20–35 years, 15%), middle-aged adults (36–50 years, 31.7%), and elders (51–80 years, 53.3%), following classifications used in previous ethnobotanical studies [18]. Of the 120 participants, 68 (56.7%) were male and 52 (43.3%) were female, reflecting the local gender roles in the collection, preparation, and use of wild edible plants.

Regarding marital status, 84 (70%) were married, 24 (20%) single, and 12 (10%) divorced. Educational attainment varied: 47% were illiterate, 20.6% had attended adult education, 19.1% completed grades 1–4, 5.9% held a diploma, and 7.4% had a university degree. Participants’ livelihoods were diverse, including livestock keepers (33.8%), farmers (30.9%), merchants (16.2%), charcoal producers or firewood sellers (10.3%), with the remainder engaged in other activities (Table 1).

### Diversity of wild edible plants in Korahe Zone

**Fig. 2:**
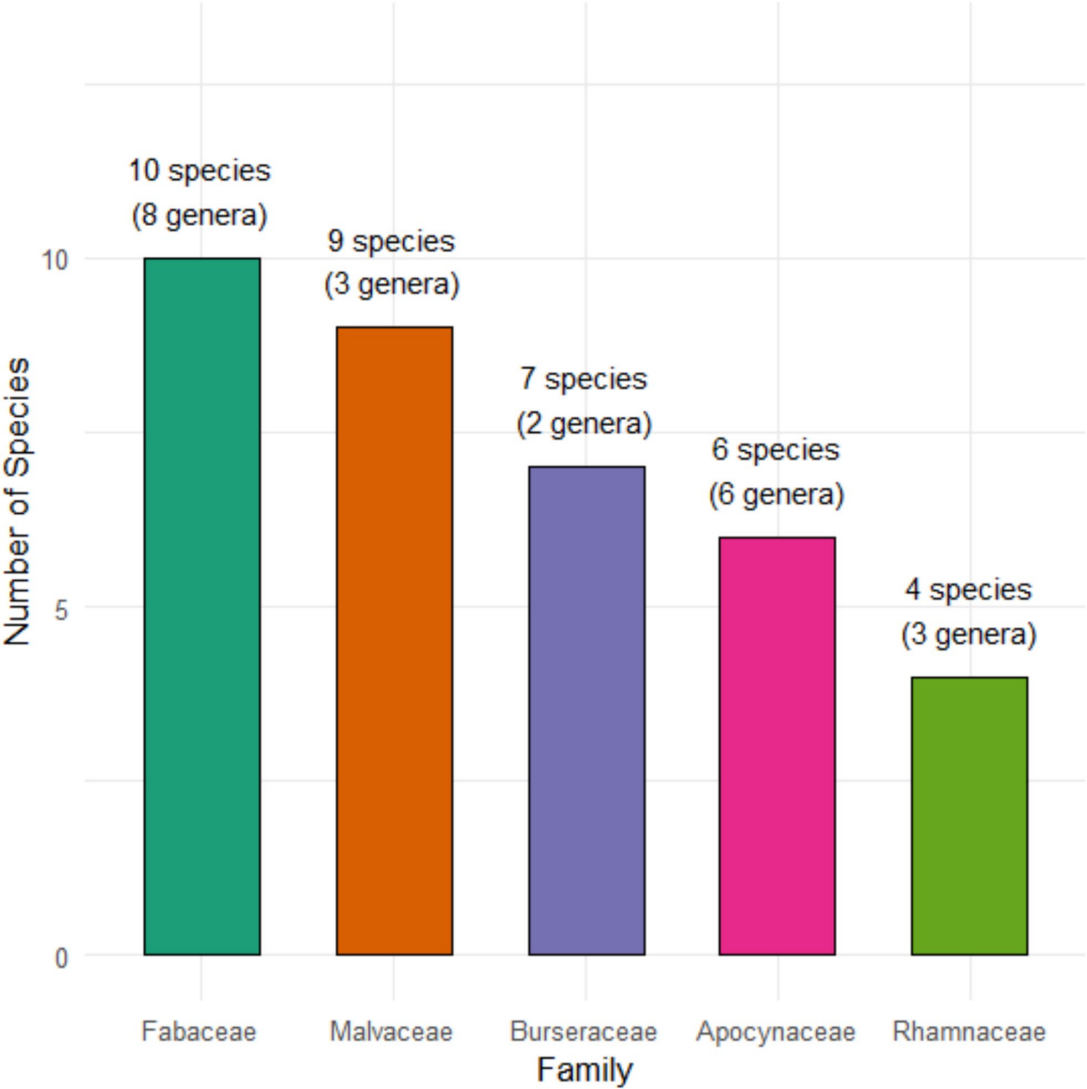
Most widely cited wild edible plants by family and genus

Table 2 A total of 57 wild edible plant (WEP) species were documented in the study area, representing 41 genera and 22 botanical families (Table 3). The Fabaceae family was the most species-rich, followed by Malvaceae, Burseraceae, Apocynaceae, and Rhamnaceae (Fig. 2), reflecting both their ecological prominence and cultural significance to local communities.

**Table 2:** Comparative analysis of study sites based on the Botanical Ethnoknowledge Index (BEI)

Study sites		N	Ms	Sg	mc	St	BEI
Study sites	Shilabo	15	9.6	36	5.7	57	0.412
	Shekosh	15	7.4	26	4.2		0.257
	K’orahe	15	8.3	29	4.9		0.311
	KebreDehar zuria	15	5.8	21	3.4		0.185
	Dumale	15	3.2	12	2.1		0.085
	Dalad	15	6.4	24	3.3		0.204
	Debe Woyin	15	9.2	32	5.2		0.355
	Biyolow	15	4.6	16	2.5		0.127

*N* = Number of participants in a particular group, *ms* = Mean number of species reported per participant in a particular group, *sg* = Total number of species reported by all participants in a particular group, *mc* = Mean number of citations per species in a particular group, *st* = Total number of species reported by all compared groups in the study, *BEI* = Botanical Ethnoknowledge Index

**Table 3:** List of wild edible plant species documented in Korahe Zone, Eastern Ethiopia, including their botanical names, families, and local uses

Family	Scientific Name	VN	Villages/sites	Local Name	Habit	PU	Preparation and Mode Consumption	PoC	MoC	SA
Amaranthaceae	*Amaranthus dubius* Mart. ex Thell	GA39	Shilabo, Debe Weyin, K’orahe, Shekosh, Kebri Dehar Zuria, Biyolow	Cayo	H	Lf	The leaves are traditionally prepared by boiling and served as a leafy vegetable	During famine	Plucking	Spring
Anacardiaceae	*Lannea triphylla* (Hochst. ex A.Rich.) Engl	GA02	Shilabo, K’orahe, Shekosh, Kebri Dehar Zuria	Wacanri, Jiidiwa	T	Fr	The ripe fruits are consumed in their raw form	At normal time	Picking	All season
	*Ozoroa insignis* Delile	GA01	Shilabo, K’orahe, Delaad, Biyolow	Biiqaga	T	Fr	The ripe fruits are consumed in their raw form	At normal time	Picking	Spring
Apocynaceae	*Carissa spinarum* L	GA12	Shilabo, Debe Weyin, Shekosh	Dhannaanowgamadow	Sh	Fr	The ripe fruits are consumed in their raw form	At normal time	Picking	Autumn
	*Ceropegia subaphylla* K.Schum	GA16	Shilabo, Debe Weyin, Shekosh, Dumale	Marooro-geel	Sh	Fr	The ripe fruits are consumed in their raw form	At normal time	Picking	Autumn
	*Cibirhiza spiculata* Thulin and Goyder	GA42	Shilabo, Debe Weyin, Shekosh, Delaad, Kebri Dehar Zuria	Doonbir	Cl	Rt	The roots are traditionally peeled, cooked, and eaten as food	At normal time	Digging	Spring
	*Echidnopsis dammanniana* E.Dammann and Sprenger	GA15	Shilabo, Debe Weyin, Shekosh, Biyolow	Carab-loaad	H	Tub	The roots or tubers are traditionally peeled, cooked, and eaten as food	At normal time	Digging	Spring
	*Edithcolea grandis* N.E.Br	GA13	Shilabo, Debe Weyin, Kebri Dehar Zuria	Xamakow	Sh	Rt	The roots are traditionally peeled, cooked, and eaten as food	At normal time	Digging	Spring
	*Glossonema boveanum* (Decne.) Decne	GA14	Shilabo, K’orahe, Delaad	Askax	Sh	Fr	The ripe fruits are consumed in their raw form	At normal time	Picking	Spring
Arecaceae	*Hyphaene reptans* Becc	GA30	Shilabo, K’orahe, Delaad	Qoona Ama Baar	T	Fr	The ripe fruits are consumed in their raw form	During famine	Picking	Spring
	*Phoenix dactylifera* L	GA55	Shilabo, K’orahe, Delaad, Kebri Dehar Zuria	Timir	S	Fr	The ripe fruits are consumed in their raw form	At normal time	Picking	Winter
Aristolochiaceae	*Hydnora abyssinica* A.Br	GA20	Debe Weyin, K’orahe, Biyolow	Diingax ana likaha	H	Fr	The ripe fruits are consumed in their raw form	During famine	Picking	Summer
Asteraceae	*Iphionopsis rotundifolia* (Oliv. and Hiern) Anderb	GA53	Debe Weyin, K’orahe, Dumale	Gabgabood	Sh	Rt	The roots are prepared by peeling and cooking before being consumed	At normal time	Digging	Winter
Balanophoraceae	*Sarcophyte sanguinea* Sparrm	GA56	Debe Weyin, K’orahe, Biyolow	Diinsi	H	Rt	The roots are traditionally peeled, cooked, and eaten as food	At normal time	Digging	Winter
Burseraceae	*Boswellia neglecta* S.Moore	GA10	Debe Weyin, K’orahe, Delaad, Kebri Dehar Zuria	Mira fur	Sh	Fr	The ripe fruits are consumed in their raw form	At normal time	Picking	Spring
	*Boswellia ogadensis* Vollesen	GA40	Shilabo, K’orahe, Shekosh, Delaad	Malmal	T	Rs	After collection, the resin is taken and consumed raw	During famine	Plucking	Winter
	*Commiphora cyclophylla* Chiov	GA11	Shilabo, K’orahe, Shekosh, Dumale	Xagar-cad	T	Fr	The ripe fruits are consumed in their raw form	At normal time	Picking	Winter
	*Commiphora myrrha* (T.Nees) Engl	GA43	Shilabo, K’orahe, Shekosh, Delaad	Dhidin	T	Rs	After collecting the resin and eat raw	At normal time	Plucking	Winter
	*Commiphora gileadensis* (L.) C.Chr	GA09	Shilabo, K’orahe, Shekosh, Kebri Dehar Zuria	Xagar madow	Sh	Fr, Rt and St	Fruit is eaten raw. The roots or tubers are traditionally peeled, cooked, and eaten as food	At normal time	Picking	Summer
	*Commiphora rostrata* Engl	GA44	Shilabo, K’orahe, Shekosh, Delaad	Jinaw	Sh	Lf	Fresh leaves are traditionally boiled and consumed as a leafy vegetable	At normal time	Plucking	Summer
	*Commiphora serrulata* Engl	GA57	Shilabo, K’orahe, Shekosh, Dumale	Bacaroor	T	Fr	The ripe fruits are consumed in their raw form	At normal time	Picking	Winter
Cactaceae	*Opuntia monacantha* Haw	GA17	Shilabo, K’orahe, Shekosh, Delaad, Kebri Dehar Zuria	Tiin	Sh	Fr	The ripe fruits are consumed in their raw form	At normal time	Picking	Spring
Capparaceae	*Capparis fascicularis* DC	GA18	Shilabo, K’orahe, Shekosh, Biyolow	Dhanaanow cad	Sh	Fr and Lf	Fresh leaves are traditionally boiled and consumed as a leafy vegetable	At normal time	Plucking	Spring
Cucurbitaceae	*Cucumis kelleri* (Cogn.) Ghebret. and Thulin	GA47	Debe Weyin, Delaad, Biyolow	Uneexo	Cl	Fr	The ripe fruits are consumed in their raw form	At normal time	Picking	Spring
Cyperaceae	*Cyperus esculentus* L. var. *leptostachyus* Boeckeler	GA19	Debe Weyin, Delaad, Kebri Dehar Zuria	Gocoso	H	Tub	The roots or tubers are traditionally peeled, cooked, and eaten as food	During famine	Digging	Summer
	*Cyperus exaltatus* Retz	GA48	Debe Weyin, Delaad, Dumale	Goon	H	Tub	The roots or tubers are traditionally peeled, cooked, and eaten as food	During famine	Digging	Summer
Euphorbiaceae	*Givotia gosai* Radcl.-Sm	GA50	Debe Weyin, Delaad, Dumale	Goosay	T	Fr	The ripe fruits are consumed in their raw form	At normal time	Picking	Spring
Fabaceae	*Acacia bussei* Harms ex Y.Sjöstedt	GA37	Debe Weyin, Delaad, Biyolow	Galool	T	Lf	Traditionally, leaves are infused in tea and consumed as a beverage	At normal time	Plucking	Summer
	*Acacia edgeworthii* T.Anderson	GA04	Shilabo, Delaad, Kebri Dehar Zuria	Quula	Sh	Fr	The ripe fruits are consumed in their raw form	At normal time	Picking	Spring
	*Acacia reficiens* Wawra and Peyr	GA38	Shilabo, Shekosh, Kebri Dehar Zuria	Qansax	T	Rs	The resin is harvested and eaten directly without processing	During famine	Plucking	Winter
	*Alysicarpus rugosus* (Willd.) DC	GA07	Shilabo, Debe Weyin, Biyolow	Farqood ama kilijow	H	Tub	The roots or tubers are traditionally peeled, cooked, and eaten as food	During famine	Digging	Summer
	*Cordeauxia edulis* Hemsl	GA45	Shilabo, Debe Weyin, Kebri Dehar Zuria	Yicib	Sh	Fr	The ripe fruits are consumed in their raw form	During famine	Picking	Autumn
	*Crotalaria dumosa* Franch	GA46	Shilabo, Debe Weyin, Delaad	Xajiin	Sh	Fr	The ripe fruits are consumed in their raw form	At normal time	Picking	Autumn
	*Delonix elata* (L.) Gamble	GA03	Shilabo, Debe Weyin, Dumale	Libi	T	Fr	The ripe fruits are consumed in their raw form	During famine	Picking	Autumn
	*Eriosema nutans* Schinz	GA06	Shilabo, K’orahe, Kebri Dehar Zuria	Kurtey	H	Tub	The roots or tubers are traditionally peeled, cooked, and eaten as food	At normal time	Digging	Summer
	*Indigofera volkensii* Taub	GA52	Shilabo, K’orahe, Delaad, Biyolow	Hajin	Sh	Rt	The roots are traditionally peeled, cooked, and eaten as food	At normal time	Digging	Winter
	*Tamarindus indica* L	GA05	Shilabo, K’orahe, Kebri Dehar Zuria	Ruqo	Sh	Fr	The ripe fruits are consumed in their raw form	At normal time	Picking	Summer
Malvaceae	*Grewia asiatica* L	GA21	Debe Weyin, K’orahe, Biyolow		Sh	Fr	The ripe fruits are consumed in their raw form	At normal time	Picking	Autumn
	*Grewia mollis* Juss	GA23	Debe Weyin, K’orahe, Delaad, Dumale	Dheebi	Sh	Fr	The ripe fruits are consumed in their raw form	At normal time	Picking	Autumn
	*Grewia penicillata* Chiov	GA24	Debe Weyin, K’orahe, Shekosh	Hohob	Sh	Fr	The ripe fruits are consumed in their raw form	At normal time	Picking	Autumn
	*Corchorus olitorius* L	GA25	Debe Weyin, K’orahe, Shekosh	Dharreraw	H	Lf	Leaves are eaten after cooked and also mixed with other foods	During famine	Plucking	Summer
	*Grewia erythraea* Schweinf	GA51	Debe Weyin, K’orahe, Shekosh, Kebri Dehar Zuria	Tukolalmis	Sh	Fr	The ripe fruits are consumed in their raw form	During famine	Picking	Autumn
	*Grewia pannosisepala* Chiov	GA26	Debe Weyin, K’orahe, Shekosh, Delaad, Biyolow	Dharrershe	Sh	Fr	The ripe fruits are consumed in their raw form	At normal time	Picking	Autumn
	*Grewia villosa* Willd	GA27	Debe Weyin, K’orahe, Shekosh	Gomosh; Kabish; Gomashaa	Sh	Fr	The ripe fruits are consumed in their raw form	At normal time	Picking	Autumn
	*Grewia tenax* (Forssk.) Fiori	GA28	Shilabo, Shekosh, Kebri Dehar Zuria	Dhafaruur	Sh	Fr	The ripe fruits are consumed in their raw form	At normal time	Picking	Spring
	*Sterculia rhynchocarpa* K.Schum	GA22	Shilabo, Shekosh, Kebri Dehar Zuria	Qaranro	Sh	Fr	The ripe fruits are consumed in their raw form	At normal time	Picking	Spring
Moraceae	*Ficus vasta* Forssk	GA29	Shilabo, K’orahe, Delaad, Biyolow	Berda	T	Fr	The ripe fruits are consumed in their raw form	During famine	Picking	Autumn
	*Ficus sycomorus* L	GA49	Shilabo, Delaad, Biyolow, Dumale	Madheedh	T	Fr	The ripe fruits are consumed in their raw form	During famine	Picking	Autumn
Moringaceae	*Moringa stenopetala* (Baker f.) Cufod	GA54	Debe Weyin, Shekosh, Kebri Dehar Zuria	Moriga	T	Lf	Fresh leaves are traditionally boiled and consumed as a leafy vegetable	At normal time	Plucking	Allseason
	*Berchemia discolor* (Klotzsch) Hemsl	GA41	Debe Weyin, Shekosh, Kebri Dehar Zuria	Dheen	T	Fr	The ripe fruits are consumed in their raw form	At normal time	Picking	Autumn
	*Phyllogeiton discolor* (Klotzsch) Herzog	GA32	Shilabo, Debe Weyin, Biyolow	Dheen	Sh	Fr	The ripe fruits are consumed in their raw form	At normal time	Picking	All season
	*Ziziphus hamur* Engl	GA33	Shilabo, Debe Weyin, Dumale	Xamur Gob	Sh	Fr	The ripe fruits are consumed in their raw form	At normal time	Picking	Autumn
	*Ziziphus mauritiana* Lam	GA31	Shilabo, Debe Weyin, Biyolow	Gob	Sh	Fr	The ripe fruits are consumed in their raw form	At normal time	Picking	Winter
Rubiaceae	*Vangueria madagascariensis* J.F.Gmel	GA34	Shilabo, Delaad, Dumale	Barruuriga	T	Fr	The ripe fruits are consumed in their raw form	At normal time	Picking	All season
Salicaceae	*Dovyalis abyssinica (A.Rich.) Warb*	GA35	Debe Weyin, Shekosh, Dumale	Ongolatz	Sh	Fr	The ripe fruits are consumed in their raw form	At normal time	Picking	Spring
Salvadoraceae	*Dobera glabra* (Forssk.) Juss. ex Poir	GA36	Debe Weyin, Shekosh, Kebri Dehar Zuria	Garas	T	Fr	The ripe fruits are consumed in their raw form	At normal time	Picking	Spring
Zygophyllaceae	*Balanites aegyptiaca* (L.) Delile	GA08	Debe Weyin, Shekosh, Delaad, Kebri Dehar Zuria	Quud	T	Fr	The ripe fruits are consumed in their raw form	At normal time	Picking	Spring

Several families, including Anacardiaceae, Arecaceae, Cyperaceae, and Moraceae, each contributed 2 species (3.51%). The remaining 12 families, Amaranthaceae, Aristolochiaceae, Asteraceae, Balanophoraceae, Cactaceae, Capparaceae, Cucurbitaceae, Euphorbiaceae, Moringaceae, Rubiaceae, Salicaceae, Salvadoraceae, and Zygophyllaceae, were represented by a single species each, accounting for 1.75% per family.

### Comparative ethnobotanical knowledge across study sites

Analysis using the Botanical Ethnoknowledge Index (BEI) revealed significant variation in ethnobotanical knowledge among Somali communities across different districts of Korahe Zone. Shilabo exhibited the highest BEI value, followed by Debe Woyin, while Dumale recorded the lowest value (Table 2). This pattern indicates notable differences in the depth and distribution of knowledge on wild edible plants (WEPs), with Shilabo and Debe Woyin communities demonstrating broader and more consistently shared knowledge.

The mean number of species reported per participant (ms) ranged from 3.2 in Dumale to 9.6 in Shilabo, highlighting that informants in Shilabo were more knowledgeable compared to other sites. Similarly, the total number of species reported by all participants (sg) was highest in Shilabo and lowest in Dumale (Table 2). The mean number of citations per species (mc) also varied, ranging from 2.1 in Dumale to 5.7 in Shilabo, indicating that plant knowledge was not only more extensive but also more consistently shared among informants in areas with higher BEI values.

Although a total of 57 WEP species were documented across all study sites, knowledge distribution was uneven. Shilabo and Debe Woyin together accounted for more than half of the reported species, whereas Kebri Dehar Zuria (21 species) and Biyolow (16 species) recorded substantially fewer taxa.

### Lifeforms of wild edible plant species

**Fig. 3:**
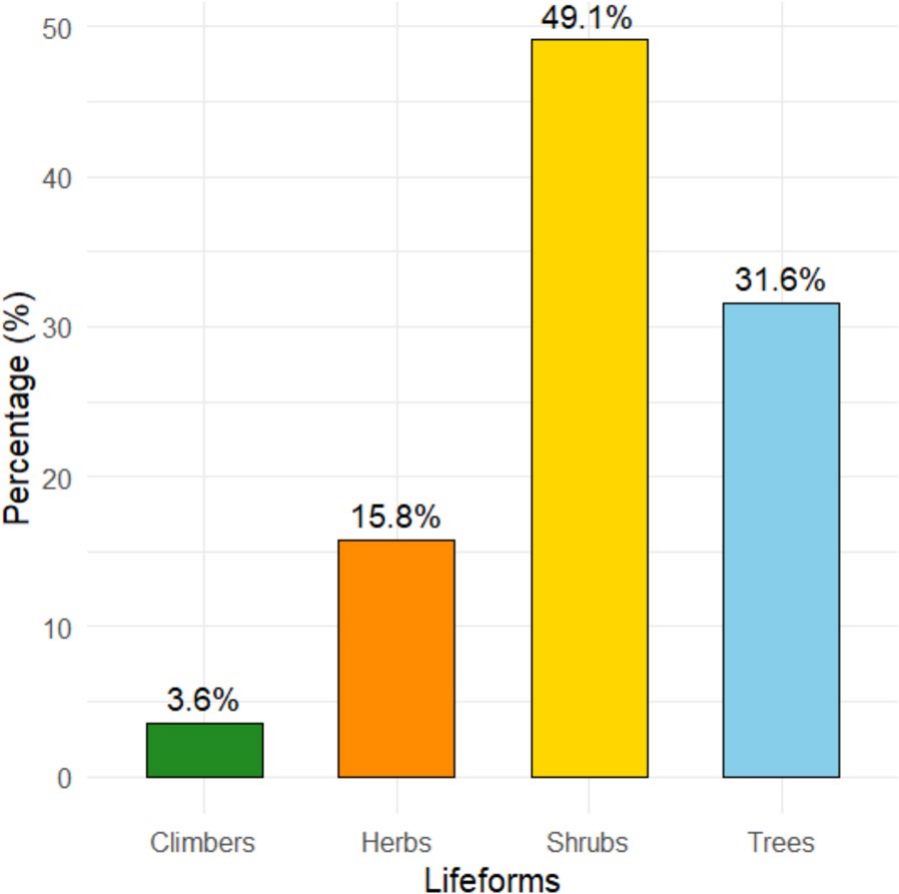
Distribution of lifeforms among wild edible plant species in Korahe Zone, Eastern Ethiopia

The study documented 57 wild edible plant (WEP) species, reflecting a wide range of ecological and morphological diversity utilized by local communities. The recorded species were categorized into four life forms: trees, shrubs, herbs, and climbers. Shrubs were the most abundant, followed by trees, herbs, and climbers (Fig. 3).

Shrubs emerged as the most important life form contributing to the local WEP resources. Representative shrub species included *Iphionopsis rotundifolia*, *Indigofera volkensii*, *Cordeauxia edulis*, *Crotalaria dumosa*, *Commiphora rostrata*, *Carissa spinarum*, and various *Grewia* species. Trees formed the second largest category, including *Ozoroa insignis*, *Lannea triphylla*, *Delonix elata*, *Acacia bussei*, *Balanites aegyptiaca*, *Ficus vasta*, and *Moringa stenopetala*.

Herbs were represented by species, such as *Amaranthus dubius*, *Sarcophyte sanguinea*, *Eriosema nutans*, *Alysicarpus rugosus*, *Cyperus esculentus*, and *Hydnora abyssinica*. Climbers were the least represented life form, including *Cucumis kelleri* and *Cibirhiza spiculata*.

### Edible parts and mode of consumption

In Korahe Zone, six categories of wild edible plant (WEP) parts were identified: fruits, tubers, roots, leaves, resin, and stems. Fruits were the most commonly consumed, comprising 66.6% of the documented species, whereas tubers, roots, and leaves each accounted for 8.7%. Resin and stems were less frequently utilized, representing 5.2% and 1.7% of species, respectively (Table 4).

**Table 4:** Informants’ consensus factor (ICF) for different wild edible plant use categories in Korahe Zone, Eastern Ethiopia

Categories	Number of taxa (Nt)	Number of use report (Nur)	Informant’s consensus factor (ICF)	RFC
Fruit	38	110	0.66	0.91
Tuber	5	92	0.95	0.77
Root	5	81	0.95	0.67
Leaf	5	64	0.93	0.53
Stem	1	42	1.00	0.35
Resin	3	53	0.96	0.44

Consumption patterns varied according to plant part. Fruits and resin were predominantly eaten raw, whereas roots, tubers, and leaves were generally cooked or mixed with other foods prior to consumption. Fruits were the most commonly used part, recorded in numerous species, including *Phoenix dactylifera*, *Ziziphus* spp., *Berchemia discolor*, *Phyllogeiton discolor*, *Vangueria madagascariensis*, *Dovyalis abyssinica*, and *Dobera glabra*.

Leaves were the second most frequently used part, occurring in species, such as *Amaranthus dubius*, *Capparis fascicularis*, *Corchorus olitorius*, and *Moringa stenopetala*. Roots were utilized in *Sarcophyte sanguinea*, *Indigofera volkensii*, *Cibirhiza spiculata*, and *Edithcolea grandis*, while tubers were reported in *Echidnopsis dammanniana*, *Cyperus esculentus*, and *Cyperus exaltatus*. Stems were used in *Commiphora gileadensis*, and resin was collected from *Acacia reficiens*, *Commiphora myrrha*, and *Boswellia ogadensis*.

Analysis of preparation and consumption revealed that ripe fruits were predominantly eaten raw, accounting for 76.5% of all observations. Roots and tubers, which require peeling and cooking, represented 17.5% of observations, reflecting the need for preparation to ensure safe consumption. Leaves, usually boiled or cooked alone or mixed with other foods, accounted for 6% of observations. Some fruits, such as *Tamarindus indica*, *Ziziphus hamur*, *Grewia asiatica*, and *Opuntia monacantha*, were also consumed raw. Informants emphasized that fruits represented the most important use of WEPs, with the highest Relative Frequency of Citation (RFC = 0.91) (Table 4).

Among use categories, fruits had the highest number of taxa (37 species) and use reports (110), though the Informant Consensus Factor (ICF) was moderate at 0.66, indicating variable agreement on specific taxa usage. In contrast, tubers and roots, each with 5 taxa, showed very high consensus (ICF = 0.95), while leaves (5 taxa, 64 use reports) had a slightly lower ICF of 0.93. Resin (3 taxa) and stems (1 taxon) recorded the highest ICF values of 0.96 and 1.00, respectively, indicating that knowledge regarding these plant parts, though limited in taxa, was consistently shared among informants.

Relative Frequency of Citation further confirmed the prominence of fruits (RFC = 0.91), followed by tubers (0.77), roots (0.67), leaves (0.53), resin (0.44), and stems (0.35), highlighting the central role of fruits in the local diet and cultural practices.

### Collection methods of wild edible plants

**Fig. 4:**
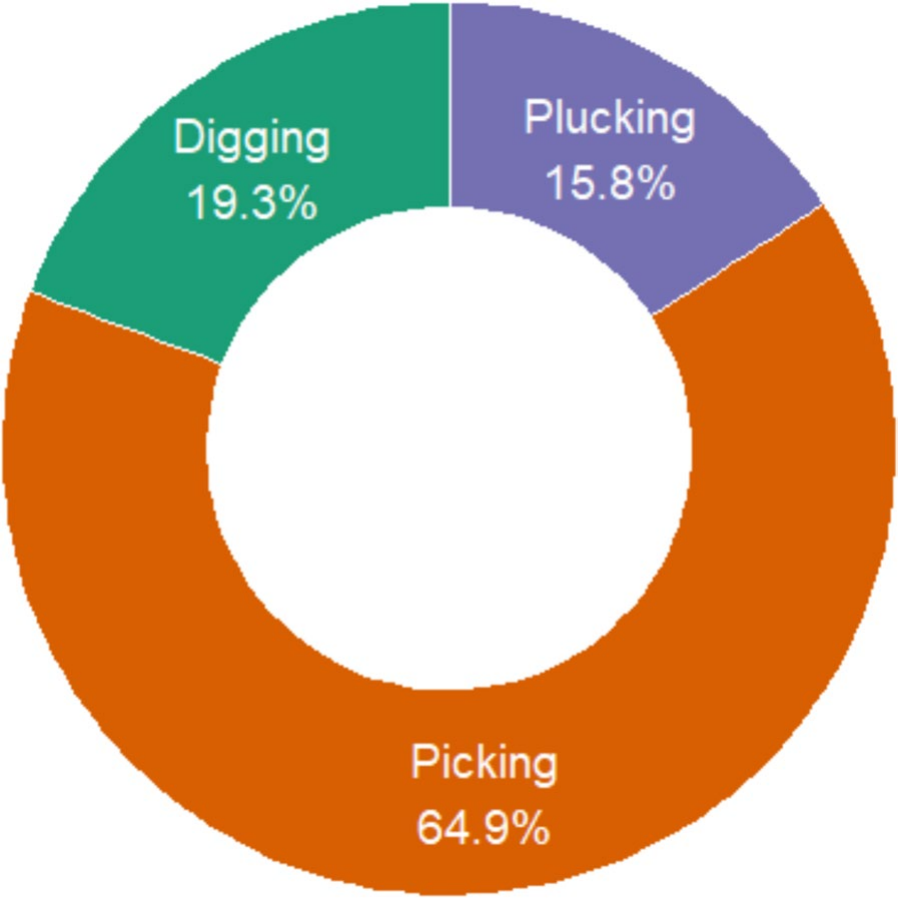
Harvesting methods of wild edible plants in Korahe Zone, Eastern Ethiopia

In Korahe Zone, local communities utilize a variety of harvesting techniques for wild and semi-wild edible plants, with the method largely depending on the specific plant part targeted. Among the 57 documented wild edible plant (WEP) species, three primary collection methods were identified: picking, plucking, and digging (Fig. 4). Picking was the most common, applied to 37 species, followed by digging for 11 species, and plucking for 9 species.

Picking was the most common method, predominantly used for fruits and other accessible plant parts without causing damage to the plant. Species collected by this method included *Ozoroa insignis*, *Ziziphus* spp., *Berchemia discolor*, *Phyllogeiton discolor*, *Vangueria madagascariensis*, *Dovyalis abyssinica*, and *Dobera glabra*.

Plucking was used for harvesting leaves, stems, or resins that could be removed carefully from the plant. Species collected by plucking included *Amaranthus dubius*, *Acacia reficiens*, *Commiphora myrrha*, *Boswellia ogadensis*, *Corchorus olitorius*, and *Moringa stenopetala*.

Digging was primarily employed for underground plant parts, such as tubers and roots. Species collected by this method included *Iphionopsis rotundifolia*, *Sarcophyte sanguinea*, *Indigofera volkensii*, *Eriosema nutans*, *Cibirhiza spiculata*, *Edithcolea grandis*, and *Cyperus exaltatus*.

### Consumption period of wild edible plants

The study documented the consumption periods of 57 wild edible plant (WEP) species, categorizing them as either consumed during normal times or primarily during famine. Analysis revealed that the majority of species (41 out of 57; 73.2%) are consumed under normal conditions, reflecting their regular inclusion in the local diet and their cultural importance as staple or supplementary foods.

Conversely, 15 species (26.8%) are primarily consumed during periods of famine, serving as emergency or supplemental food resources when conventional food sources are scarce. These famine foods play a critical role in sustaining communities during times of food insecurity, complementing the routine nutrition provided by other WEPs.

Species consumed under normal conditions include *Ozoroa insignis*, *Iphionopsis rotundifolia*, *Sarcophyte sanguinea*, *Tamarindus indica*, *Balanites aegyptiaca*, *Sterculia rhynchocarpa*, *Grewia penicillata*, *Moringa stenopetala*, *Phoenix dactylifera*, *Ziziphus mauritiana*, *Berchemia discolor*, *Vangueria madagascariensis*, *Dovyalis abyssinica*, and *Dobera glabra*.

Famine-associated species, consumed primarily during times of scarcity, include *Amaranthus dubius*, *Delonix elata*, *Acacia reficiens*, *Cordeauxia edulis*, *Boswellia ogadensis*, *Cyperus esculentus*, *Hydnora abyssinica*, *Corchorus olitorius*, *Ficus vasta*, and *Hyphaene reptans*.

### Seasonal availability of wild edible plants

**Fig. 5:**
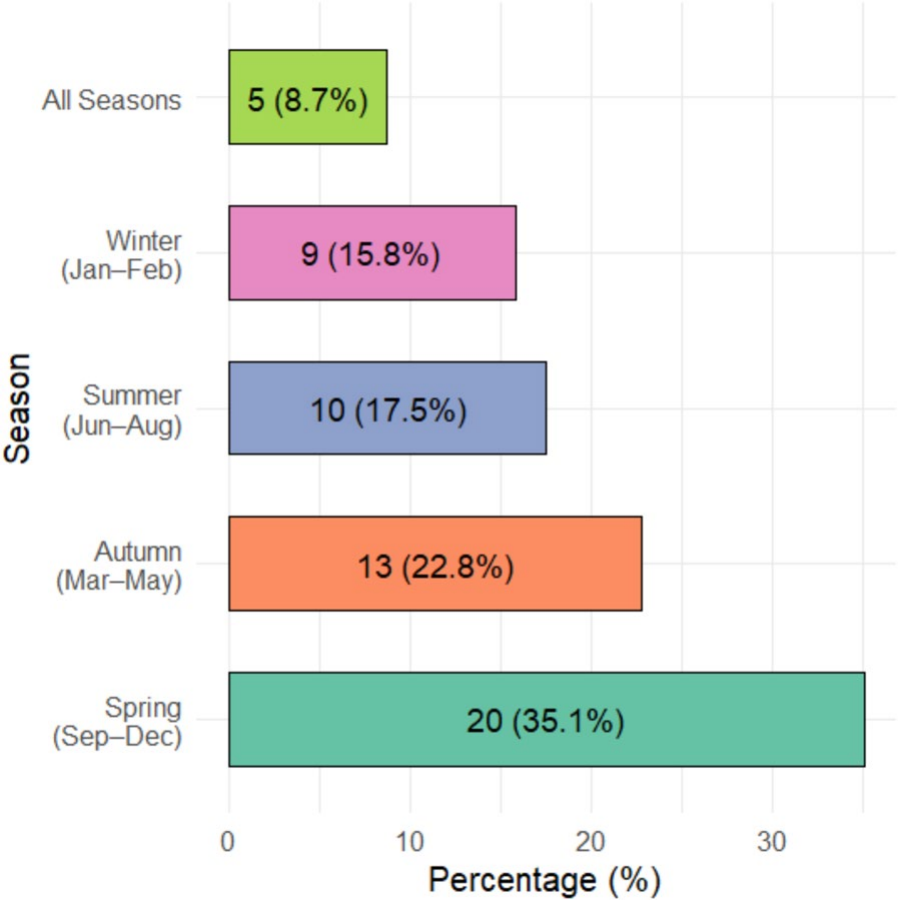
Seasonal availability of wild edible plants in Korahe Zone, Eastern Ethiopia

In Korahe Zone, the climate is broadly divided into dry (November–April) and rainy (May–October) seasons. Wild edible plants (WEPs) were available throughout the year, although their abundance and diversity varied across seasons. The analysis indicated that the greatest number of wild edible plant (WEP) species was collected during spring (September–December), followed by autumn (March–May), summer (June–August), and winter (January–February). Only a small proportion of species was available year-round (Fig. 5).

Spring represents the peak season for many WEPs, including *Amaranthus dubius*, *Ozoroa insignis*, *Sterculia rhynchocarpa*, *Grewia tenax*, *Hyphaene reptans*, *Dovyalis abyssinica*, and *Dobera glabra*.

Species available in summer include *Acacia bussei*, *Tamarindus indica*, *Eriosema nutans*, *Alysicarpus rugosus*, *Commiphora gileadensis*, *Cyperus esculentus*, and *Corchorus olitorius*.

Autumn species consist of *Delonix elata*, *Cordeauxia edulis*, *Crotalaria dumosa*, *Carissa spinarum*, *Ceropegia subaphylla*, *Grewia pannosisepala*, *Ficus sycomorus*, and *Ziziphus hamur*.

Winter species include *Iphionopsis rotundifolia*, *Sarcophyte sanguinea*, *Acacia reficiens*, *Indigofera volkensii*, *Commiphora myrrha*, *Boswellia ogadensis*, and *Commiphora cyclophylla*. Several species were available throughout the year, such as *Lannea triphylla*, *Moringa stenopetala*, *Phyllogeiton discolor*, and *Vangueria madagascariensis*, highlighting their potential as stable, continuous food resources. Community members also preserved and stored some species to ensure availability during off-peak seasons, including *Eriosema nutans*, *Alysicarpus rugosus*, *Echidnopsis dammanniana*, and *Edithcolea grandis*.

Relative Frequency of Citation (RFC) analysis indicated that WEPs are more significant during the rainy season (RFC = 0.49) than in the dry season (RFC = 0.36) or throughout the year (RFC = 0.10), emphasizing the seasonal dependence of wild food resources in local dietary practices.

### Marketability of wild edible plants in Korahe Zone

Market surveys conducted in the study area revealed that several wild edible plants (WEPs) are sold in local markets, providing an additional source of income for households. Five species were identified as actively marketable: *Corchorus olitorius* L., *Delonix elata* (L.) Gamble, *Grewia penicillata* Chiov., *Ziziphus mauritiana* Lam., and *Tamarindus indica* L. (Table 5).

**Table 5:** Marketability of wild edible plants in Korahe Zone, Eastern Ethiopia

Scientific Name	Part Sold	Seasonality	Relative Market Value
*Balanites aegyptiaca* (L.) Delile	Fruit	Spring	High
*Cordeauxia edulis* Hemsl	Fruit	Autumn	High
*Phoenix dactylifera* L	Fruit	Winter	High
*Amaranthus dubius* Mart. ex Thell	Leaves	Spring	Medium
*Moringa stenopetala* (Baker f.) Cuf	Leaves	All season	Medium
*Lannea triphylla* (Hochst. ex A.Rich.) Engl	Fruit	All season	Medium
*Cyperus esculentus* L. var. *leptostachyus* Boeckeler	Tuber	Summer	Medium
*Echidnopsis dammanniana* E.Dammann and Sprenger	Tuber	Spring	Medium
*Tamarindus indica* L	Fruit	Summer	Medium
*Grewia tenax* (Forssk.) Fiori	Fruit	Spring	Medium
*Ficus sycomorus* L	Fruit	Autumn	Medium
*Capparis fascicularis* DC	Leaves/Fruit	Spring	Low
*Opuntia monacantha* Haw	Fruit	Spring	Low
*Corchorus olitorius* L	Leaves	Summer	Low
*Hyphaene reptans* Becc	Fruit	Spring	Low
*Corchorus olitorius* L	Leave	Summer	High
*Delonix elata* (L.) Gamble	Fruit	Autumn	High
*Grewia penicillata* Chiov	Fruit	Autumn	High
*Ziziphus mauritiana* Lam	Fruit	Winter	High

High (> 100 ETB/kg), Medium (50–99 ETB/kg), Low (< 50 ETB/kg),* ETB* = Ethiopian Birr

*Corchorus olitorius* (local name: Dhareeraw) was primarily sold by women, with leaves marketed in small batches at prices ranging from 20 to 80 Ethiopian birr per unit. *Tamarindus indica* (Ruqo) was sold by both men and women, with fruits offered individually or in packaged forms, typically priced between 30 and 50 ETB per cup. *Grewia penicillata* (Hohob) fruits were sold by women in cups, ranging from 10 to 70 ETB per unit, while *Ziziphus mauritiana* (Gob) fruits were marketed by women at 10–45 ETB per cup.

Several species demonstrated notable market potential due to high local demand, palatability, and nutritional and economic value. Among the most commercially valuable were *Ziziphus mauritiana*, *Corchorus olitorius*, *Balanites aegyptiaca*, *Cordeauxia edulis*, and *Phoenix dactylifera*, which are predominantly traded for their fruits during spring, autumn, and winter.

Other species, including *Amaranthus dubius*, *Moringa stenopetala*, *Lannea triphylla*, *Cyperus esculentus*, *Echidnopsis dammanniana*, *Tamarindus indica*, *Grewia tenax*, and *Ficus sycomorus*, were moderately traded. Their marketability largely depended on seasonal availability, with some species, such as *Moringa stenopetala* and *Lannea triphylla*, available year-round, providing a reliable source of income. These species were mainly sold for their leaves, fruits, or tubers, offering both nutritional and economic benefits to local households.

Conversely, species such as *Capparis fascicularis*, *Opuntia monacantha*, *Corchorus olitorius*, and *Hyphaene reptans* had lower market value, reflecting limited demand or smaller quantities sold.

### Adverse effects associated with consumption of wild edible plants

While wild edible plants (WEPs) provide significant dietary and medicinal benefits, certain species may cause adverse effects if consumed inappropriately or in excessive amounts. According to local informants, mild gastrointestinal disturbances were observed for some leafy vegetables and tuberous species, including *Amaranthus dubius*, *Eriosema nutans*, *Alysicarpus rugosus*, *Cyperus esculentus*, and *Cyperus exaltatus*. These effects were mainly attributed to high fiber content or the presence of secondary metabolites. Consumption of certain fruits, such as *Cordeauxia edulis* and *Delonix elata*, was occasionally associated with mild stomach upset when eaten in large quantities (Table 6).

**Table 6:** Reported potential adverse effects associated with consumption of wild edible plants in Korahe Zone, Eastern Ethiopia

Scientific name	Reported/potential adverse effects
*Acacia bussei* Harms ex Y.Sjöstedt	Rare irritation in oral cavity if excessive
*Acacia edgeworthii* T.Anderson	Occasional digestive discomfort
*Amaranthus dubius* Mart. ex Thell	Mild gastrointestinal upset
*Balanites aegyptiaca* (L.) Delile	Bitterness if not properly prepared
*Berchemia discolor* (Klotzsch) Hemsl	Rare digestive discomfort
*Boswellia ogadensis* Vollesen	Oral irritation; mild digestive upset
*Capparis fascicularis* DC	Leaves may cause minor irritation
*Carissa spinarum* L	Mild stomach upset if overconsumed
*Cibirhiza spiculata* Thulin and Goyder	Toxic if eaten raw; requires proper preparation
*Commiphora gileadensis* (L.) C.Chr	Resin may cause oral irritation
*Commiphora myrrha* (T.Nees) Engl	Irritation of mouth and throat if resin overused
*Commiphora rostrata* Engl	Rare mild oral irritation
*Corchorus olitorius* L	Rare digestive upset during overconsumption
*Cucumis kelleri* (Cogn.) Ghebret. andThulin	Mild stomach upset when overconsumed
*Delonix elata* (L.) Gamble	Mild gastrointestinal discomfort
*Dovyalis abyssinica* (A.Rich.) Warb	Mild gastrointestinal upset
*Edithcolea grandis* N.E.Br	Mild gastrointestinal upset
*Eriosema nutans* Schinz	Mild digestive upset if eaten raw
*Ficus vasta* Forssk	Gastrointestinal upset if eaten in large quantities
*Grewia pannosisepala* Chiov	Rare digestive discomfort
*Grewia villosa* Willd	Mild digestive upset
*Hydnora abyssinica* A.Br	Digestive upset if overconsumed
*Hyphaene reptans* Becc	Mild gastrointestinal upset
*Indigofera volkensii* Taub	Mild digestive discomfort
*Lannea triphylla* (Hochst. ex A.Rich.) Engl	Occasional mild stomach upset
*Moringa stenopetala* (Baker f.) Cuf	Rare mild digestive upset
*Opuntia monacantha* Haw	Rare mild digestive upset
*Phoenix dactylifera* L	Rare stomach discomfort
*Phyllogeiton discolor* (Klotzsch) Herzog	Rare mild stomach upset
*Sarcophyte sanguinea* Sparrm	Potential toxicity if improperly prepared
*Tamarindus indica* L	Rare gastrointestinal upset
*Vangueria madagascariensis* J.F.Gmel	Rare digestive discomfort
*Ziziphus mauritiana* Lam	Mild gastrointestinal upset

Allergic reactions or irritation were reported for a few species containing resins or latex, including *Commiphora myrrha*, *Acacia reficiens*, and *Boswellia ogadensis*. These reactions were typically localized to the oral cavity or skin during handling or chewing.

Potential toxicity was a concern for certain tubers and underground parts, such as *Iphionopsis rotundifolia*, *Sarcophyte sanguinea*, *Cibirhiza spiculata*, and *Echidnopsis dammanniana*, when consumed raw or without proper processing. Informants emphasized that these species require cooking or other forms of treatment to reduce toxic compounds and ensure safe consumption.

### Preference ranking of wild edible plants

Preference ranking was conducted to identify the most favored wild edible plants (WEPs) based on key criteria including taste, nutritional value, availability, and ease of collection. Informants assigned scores to selected species, with higher scores indicating greater preference.

The analysis revealed that *Cordeauxia edulis*, *Balanites aegyptiaca*, *Amaranthus dubius*, *Moringa stenopetala*, and *Phoenix dactylifera* were the most preferred species. These plants are highly valued due to their palatability, nutritional content, and either year-round or seasonal availability (Table 7).

**Table 7:** Preference ranking of wild edible plants in Korahe Zone, Eastern Ethiopia, based on taste, nutritional value, availability, and ease of collection

Rank	Scientific name	Score	Key attributes
1	*Cordeauxia edulis* Hemsl	85	Highly palatable fruit, high nutritional value, culturally important
2	*Balanites aegyptiaca* (L.) Delile	82	Nutritious fruit, multipurpose use, widely available
3	*Amaranthus dubius* Mart. ex Thell	77	Leafy vegetable, easy to harvest, commonly consumed
4	*Moringa stenopetala* (Baker f.) Cuf	74	Leaves highly nutritious, year-round availability
5	*Phoenix dactylifera* L	69	Sweet fruit, widely used, long shelf life
6	*Lannea triphylla* (Hochst. ex A.Rich.) Engl	65	Fruit edible year-round, multipurpose use
7	*Ziziphus mauritiana* Lam	62	Palatable fruit, culturally valued
8	*Ficus sycomorus* L	58	Fruit widely used locally, seasonal availability
9	*Commiphora myrrha* (T.Nees) Engl	56	Fruit and resin used, medicinal and food value
10	*Grewia tenax* (Forssk.) Fiori	52	Edible fruit, used seasonally
11	*Hyphaene reptans* Becc	49	Edible fruit, famine food, multipurpose
12	*Delonix elata* (L.) Gamble	42	Fruit edible mainly during scarcity periods
13	*Cyperus esculentus* L. var. *leptostachyus* Boeckeler	36	Tubers consumed mainly during famine
14	*Echidnopsis dammanniana* E.Dammann and Sprenger	32	Tubers consumed as emergency food
15	*Corchorus olitorius* L	30	Leafy vegetable, mainly famine usage

Species such as *Ziziphus mauritiana*, *Ficus sycomorus*, *Lannea triphylla*, and *Commiphora myrrha* received moderate scores, reflecting their cultural and dietary significance, but slightly lower preference due to factors, such as taste or limited seasonal availability.

The least preferred species included some tuberous and resinous plants, such as *Cyperus esculentus*, *Echidnopsis dammanniana*, and *Boswellia ogadensis*. These species are primarily consumed during famine periods or require extensive processing to remove bitterness or potential toxins.

### Direct matrix ranking of multi-purpose wild edible plants

Direct Matrix Ranking (DMR) was employed to evaluate the relative importance of multi-purpose wild edible plants (WEPs) based on their diverse uses, including food, medicinal value, fuel, construction, fodder, and other cultural or economic applications. Informants assigned scores to each species within these use categories, and cumulative scores were used to rank species according to overall utility.

Species such as *Balanites aegyptiaca*, *Cordeauxia edulis*, *Commiphora myrrha*, *Boswellia ogadensis*, and *Moringa stenopetala* scored highest across multiple use categories (Table 8). These plants not only provide edible parts, such as fruits, leaves, and tubers, but are also utilized for medicinal purposes, fuelwood, construction materials, fodder, and cultural practices. Their high cumulative scores indicate that they are integral to local livelihoods and are heavily relied upon by the community.

**Table 8:** Average direct matrix ranking (DMR) of multi-purpose wild edible plants in Korahe Zone, Eastern Ethiopia, based on food, medicinal, fuel, construction, fodder, and other cultural or economic uses

Scientific name	FD	MD	FU	CN	FO	OU	TS	Rank
*Balanites aegyptiaca* (L.) Delile	5	4	4	3	4	3	23	1st
*Cordeauxia edulis* Hemsl	5	3	3	3	3	3	20	2nd
*Commiphora myrrha* (T.Nees) Engl	4	5	3	2	2	4	20	3rd
*Moringa stenopetala* (Baker f.) Cuf	5	4	2	3	3	2	19	4th
*Phoenix dactylifera* L	5	3	3	4	2	2	19	5th
*Lannea triphylla* (Hochst. ex A.Rich.) Engl	4	3	3	4	3	2	19	6th
*Boswellia ogadensis* Vollesen	3	5	4	3	2	2	19	7th
*Commiphora cyclophylla* Chiov	4	4	3	3	2	2	18	8th
*Hyphaene reptans* Becc	5	3	2	3	3	2	18	9th
*Ziziphus mauritiana* Lam	5	3	2	3	2	2	17	10th
*Ficus sycomorus* L	5	2	3	4	2	1	17	11th
*Grewia tenax* (Forssk.) Fiori	4	3	2	2	2	2	15	12th
*Delonix elata* (L.) Gamble	4	2	2	2	1	2	13	13th
*Cyperus esculentus* L. var. *leptostachyus* Boeckeler	4	1	2	1	1	1	10	14th
*Echidnopsis dammanniana* E.Dammann and Sprenger	4	1	1	1	1	1	9	15th
TS	66	46	39	41	33	31		
R	1st	2nd	4th	3rd	5th	6th		

*R*** = **Rank, *FD*** = **Food, *MD*** = **Medicine, *FU*** = **Fuel, *CN*** = **Construction, *FO*** = **Fodder, *OU*** = **other uses, *TS*** = **Total Score

Other species, including *Amaranthus dubius*, *Ziziphus mauritiana*, *Lannea triphylla*, and *Ficus sycomorus*, received moderate rankings. These plants are primarily valued for their food use but have fewer additional applications compared to the top-ranking species, highlighting their narrower functional role in local ethnobotanical practices.

### Most popular wild edible plants

Among the 57 documented wild edible plant (WEP) species, certain plants emerged as highly popular due to their frequent use, accessibility, palatability, and cultural significance. Popularity was evaluated using the Relative Frequency of Citation (RFC), informant consensus, and reported usage patterns.

Fruits of several species were among the most widely used and preferred by the community. These include *Cordeauxia edulis*, *Balanites aegyptiaca*, *Phoenix dactylifera*, *Ziziphus mauritiana*, *Lannea triphylla*, *Ficus sycomorus*, and *Vangueria madagascariensis* (Table 9). These species are highly valued for their taste, nutritional content, and, in some cases, year-round availability.

**Table 9:** Most popular wild edible plants (WEPs) in the Korahe Zone, Eastern Ethiopia, based on relative frequency of citation (RFC), informant consensus, and reported usage patterns

Scientific name	Key usage	RFC	Rank
*Amaranthus dubius* Mart. ex Thell	Leafy vegetable, widely consumed	0.91	1st
*Balanites aegyptiaca* (L.) Delile	Fruit edible, multipurpose, famine food	0.88	2nd
*Cordeauxia edulis* Hemsl	Fruit, famine food, high cultural significance	0.86	3rd
*Moringa stenopetala* (Baker f.) Cuf	Leaves consumed regularly, nutritionally rich	0.83	4th
*Phoenix dactylifera* L	Fruit widely used, culturally important	0.81	5th
*Ziziphus mauritiana* Lam	Fruit consumed seasonally, palatable	0.79	6th
*Lannea triphylla* (Hochst. ex A.Rich.) Engl	Fruit edible year-round, multipurpose use	0.78	7th
*Ficus sycomorus* L	Fruit consumed locally, seasonal availability	0.76	8th
*Commiphora myrrha* (T.Nees) Engl	Fruit and resin used, medicinal and food value	0.74	9th
*Grewia tenax* (Forssk.) Fiori	Edible fruit, used seasonally	0.72	10th
*Delonix elata* (L.) Gamble	Fruit consumed mainly during scarcity periods	0.70	11th
*Cyperus esculentus* L. var. *leptostachyus* Boeckeler	Tubers consumed mainly during famine	0.68	12th
*Echidnopsis dammanniana* E.Dammann and Sprenger	Tubers consumed as emergency food	0.66	13th
*Corchorus olitorius* L	Leafy vegetable, mainly famine usage	0.65	14th
*Hyphaene reptans* Becc	Fruit consumed seasonally, famine food	0.63	15th

Leafy vegetables, such as *Amaranthus dubius*, *Corchorus olitorius*, and *Moringa stenopetala*, were also frequently cited. These species are commonly harvested and consumed during normal periods, providing essential vitamins and minerals and playing a key role in local diets.

Roots and tubers of species including *Eriosema nutans*, *Alysicarpus rugosus*, *Cyperus esculentus*, and *Echidnopsis dammanniana* are particularly important during famine periods, underscoring their role as emergency food resources.

In addition, resin-producing species, such as *Commiphora myrrha* and *Boswellia ogadensis*, were widely recognized not only for their nutritional value but also for their medicinal applications, reflecting their multifaceted importance within the community.

### Newly identified wild edible plants in Korahe Zone, Somali Region, Ethiopia

Wild edible plants (WEPs) are vital components of food systems in arid and semi-arid regions of Ethiopia, particularly in the Somali Region, where agro-pastoral livelihoods rely heavily on natural vegetation for nutrition and resilience against food shortages. Despite their importance, ethnobotanical documentation of WEPs in the Korahe Zone has been limited. By comparing a reference checklist of 57 Ethiopian WEPs with field surveys and community reports, this study identified several species newly recognized as edible resources in Korahe.

The findings revealed nine species that are locally utilized for food but had not been formally documented in this zone. These include *Cordeauxia edulis*, whose seeds are consumed raw or roasted and are culturally significant as a famine food; *Boswellia neglecta*, from which gum and resin are occasionally chewed by children as a sweetener; and *Commiphora myrrha* and *Commiphora serrulata*, valued for their resin, sometimes chewed or used as flavoring, with young shoots of *C. serrulata* eaten as snacks. *Dobera glabra* was reported as an important drought food, with fruits consumed during periods of scarcity, while *Ziziphus hamur* provides fruits eaten fresh and considered a key dryland resource. Other widely gathered fruits include those of *Grewia tenax* and *Carissa spinarum*, consumed either fresh or dried for storage. Finally, *Hyphaene reptans* contributes fruits and edible young shoots, with the palm heart occasionally harvested.

The identification of these species expands the documented ethnobotanical range of wild food plants in the Somali Region. Many of them, such as *Cordeauxia edulis* and *Dobera glabra*, are already recognized across the Horn of Africa as crucial famine foods, reflecting the importance of perennial woody plants in sustaining households during drought. The inclusion of *Cordeauxia edulis*, a species listed as vulnerable on the IUCN Red List, further emphasizes the need for targeted conservation measures and sustainable management to ensure the continued availability of this important food resource.

### Implications of using wild edible plants in Korahe Zone

The 57 documented wild edible plant (WEP) species play a critical role in ensuring food security in the Korahe Zone, particularly during periods of drought or famine. Many species, including *Cyperus esculentus*, *Echidnopsis dammanniana*, and *Amaranthus dubius*, provide essential nutrients and calories when conventional food sources are limited (Table 10). Seasonal fruits and tubers serve as supplementary food sources, enhancing dietary diversity and reducing household vulnerability to food shortages.

**Table 10:** Top 20 wild edible plants in Korahe Zone, selected for their combined contribution to food security, health, cultural significance, and environmental sustainability

Scientific name	Food security	Health and nutrition	Cultural use	Environmental protection
*Balanites aegyptiaca* (L.) Delile	Fruit source	Rich in vitamins and minerals	Ceremonial use	Soil stabilization, biodiversity
*Cordeauxia edulis* Hemsl	Fruit during famine	Rich in carbohydrates and minerals	Cultural consumption	Agroforestry support
*Phoenix dactylifera* L	Fruit supplement	Vitamins and minerals	Cultural and dietary use	Tree cover, erosion control
*Amaranthus dubius* Mart. ex Thell	Leafy vegetable during scarcity	Rich in vitamins and minerals	Traditional diet	Home garden cultivation
*Moringa stenopetala* (Baker f.) Cufod	Leaves year-round	Rich in vitamins and minerals	Traditional consumption	Agroforestry, soil enrichment
*Lannea triphylla* (Hochst. ex A.Rich.) Engl	Year-round fruit source	Micronutrient-rich	Ceremonial use	Soil stabilization
*Grewia tenax* (Forssk.) Fiori	Fruit supplement	Vitamins and minerals	Traditional use	Shrubland support
*Ficus sycomorus* L	Fruit	Vitamins and minerals	Ceremonial and dietary use	Habitat and soil support
*Tamarindus indica* L	Fruit supplement	High nutrient content	Cultural recipes	Shade and habitat diversity
*Cyperus esculentus* L. var. *leptostachyus* Boeckeler	Tuber during famine	Energy source	Minimal cultural use	Wetland soil stabilization
*Commiphora gileadensis* (L.) C.Chr	Fruit, root, stem	Medicinal and nutritional	Cultural medicine	Shrubland and ecosystem support
*Commiphora myrrha* (T.Nees) Engl	Resin use	Medicinal properties	Ritual/traditional use	Shrubland stability
*Balanites aegyptiaca* (L.) Delile	Fruit/leaf	Nutritional	Cultural use	Soil conservation
*Tamarindus indica* L	Fruit supplement	Nutritional	Cultural ceremonies	Agroforestry support
*Ficus vasta* Forssk	Fruit	Nutritional	Cultural rituals	Tree cover and soil protection
*Hyphaene reptans* Becc	Fruit	Nutritional	Minimal cultural use	Soil stabilization
*Acacia bussei* Harms ex Y.Sjöstedt	Leaf consumption	Nutrient source	Traditional diet	Nitrogen fixation
*Cibirhiza spiculata* Thulin and Goyder	Root consumption	Energy-dense	Limited cultural use	Soil enrichment
*Grewia mollis* Juss	Fruit	Nutritional	Cultural use	Shrubland support
*Commiphora serrulata* Engl	Fruit	Nutritional	Local use	Soil and habitat protection

Beyond their role in food security, WEPs contribute significantly to nutrition and overall health. Leaves of species such as *Moringa stenopetala* and *Amaranthus dubius* are rich in vitamins, minerals, and antioxidants, supporting immune function and preventing malnutrition. Fruits from *Balanites aegyptiaca*, *Cordeauxia edulis*, and *Grewia tenax* provide essential carbohydrates and micronutrients, while tubers and roots supply energy-dense foods during periods of scarcity. Several species also possess medicinal properties, complementing traditional healthcare practices and reducing dependence on pharmaceutical products.

Wild edible plants hold substantial cultural and traditional value within local communities. They are integrated into rituals, ceremonies, and traditional diets, and knowledge of their use is transmitted across generations. Species such as *Tamarindus indica*, *Ficus sycomorus*, and *Commiphora myrrha* serve not only as food sources but also as symbolic elements in cultural practices, reinforcing social cohesion and preserving ethnobotanical heritage.

The sustainable utilization of WEPs also supports environmental conservation. Many species, including *Balanites aegyptiaca* and *Lannea triphylla*, contribute to soil stabilization, habitat diversity, and carbon sequestration. When harvested using community-led, well-managed practices, these plants reduce pressure on agricultural lands while promoting biodiversity conservation. Furthermore, the cultivation of high-demand species in home gardens or agroforestry systems enhances ecosystem resilience, ensuring continued availability of local food resources while maintaining ecological balance.

### Conservation and management of wild edible plants

Wild edible plants (WEPs) in Korahe Zone play an essential role in food security, nutrition, and cultural heritage. However, their sustainability is increasingly threatened by a combination of anthropogenic and environmental pressures (Table 11).

**Table 11:** Perceived threats to wild edible plants in Korahe Zone

Threat category	Description	Affected species (examples)
Overharvesting	Excessive collection of edible parts (fruits, leaves, tubers, resin)	*Cordeauxia edulis*, *Balanites aegyptiaca*, *Amaranthus dubius*, *Moringa stenopetala*
Habitat loss/degradation	Conversion of forests and grasslands to agriculture, urbanization	*Lannea triphylla*, *Vangueria madagascariensis*, *Commiphora myrrha*
Climate change/erratic rainfall	Affects flowering, fruiting, and tuber formation	*Cyperus esculentus*, *Echidnopsis dammanniana*, *Hydnora abyssinica*
Grazing/trampling by livestock	Damage to herbs and shrubs, reducing regeneration	*Amaranthus dubius*, *Eriosema nutans*, *Corchorus olitorius*
Invasive species/pests	Competition for resources or damage by herbivores or insects	*Balanites aegyptiaca*, *Phoenix dactylifera*, *Commiphora myrrha*
Limited propagation/low regeneration	Species with slow growth or low seed dispersal	*Boswellia ogadensis*, *Commiphora cyclophylla*, *Hyphaene reptans*
Charcoal and fire wood making	Damage to trees and shrubs, reducing regeneration	*Balanites aegyptiaca*, *Boswellia ogadensis*, *Commiphora cyclophylla*

Overharvesting was identified as a primary concern, especially for highly valued species, such as *Cordeauxia edulis*, *Balanites aegyptiaca*, *Amaranthus dubius*, and *Moringa stenopetala*. Intensive collection of fruits, leaves, roots, and resins without sufficient regeneration has led to declining populations. Habitat loss and degradation from agricultural expansion, deforestation, and urbanization were also reported, particularly affecting tree and shrub species, such as *Lannea triphylla*, *Vangueria madagascariensis*, and *Commiphora myrrha*. Informants noted that the clearing of natural vegetation diminishes both the spatial distribution of WEPs and the ecological niches they occupy.

Climate variability further compounds these threats. Erratic rainfall and prolonged droughts disrupt flowering, fruiting, and tuber formation in species dependent on seasonal precipitation, including *Cyperus esculentus*, *Echidnopsis dammanniana*, and *Hydnora abyssinica*. Heavy grazing and trampling by livestock were also recognized as major constraints, especially for herbaceous and shrub species, such as *Amaranthus dubius*, *Eriosema nutans*, and *Corchorus olitorius*, which are highly susceptible to regeneration failure.

The spread of invasive species and pests exacerbates the situation. Aggressive invaders such as *Parthenium hysterophorus* L. and *Prosopis juliflora* (Sw.) DC. displace native vegetation, alter soil conditions, and compete for limited resources, further reducing the availability and quality of edible plants (Fig. S5).

Screening of the 57 documented WEPs against the IUCN Red List revealed that three species are globally threatened. *Boswellia ogadensis* is listed as Critically Endangered (CR) due to overexploitation and habitat loss, while *Cordeauxia edulis* (Yeheb) and *Commiphora cyclophylla* are categorized as Vulnerable (VU) (Table 12). Other widely used species, such as *Tamarindus indica*, *Balanites aegyptiaca*, *Carissa spinarum*, *Grewia tenax*, and *Vangueria madagascariensis*, are currently assessed as Least Concern (LC) or Not Evaluated (NE), yet remain under pressure from overharvesting, deforestation, and grazing.

**Table 12:** IUCN red list status of wild edible plants in Korahe Zone, Somali Region, Ethiopia

Scientific name	IUCN status	Notes on threats and importance
*Boswellia ogadensis*	Critically Endangered (CR)	Endemic to Somalia–Ethiopia drylands; overexploitation and habitat loss threaten its survival
*Cordeauxia edulis*	Vulnerable (VU)	Endemic leguminous shrub; edible seeds are a critical food source in arid regions; unsustainable seed collection and land degradation are major concerns
*Commiphora cyclophylla*	Vulnerable (VU)	Myrrh-producing species; restricted to arid Ethiopia and Somalia; overharvesting of resin and habitat disturbance are significant threats
*Tamarindus indica*	Least Concern (LC)	Widely used for its edible fruits; faces pressures from deforestation and overgrazing
*Balanites aegyptiaca*	Least Concern (LC)	Provides edible fruits; habitat degradation and overharvesting are concerns
*Carissa spinarum*	Least Concern (LC)	Edible fruits; overgrazing and land use change pose threats
*Grewia tenax*	Least Concern (LC)	Fruits consumed widely; habitat loss and unsustainable harvesting are issues
*Vangueria madagascariensis*	Least Concern (LC)	Edible fruits; faces threats from land use change and overgrazing
*Ziziphus spina-christi*	Not Evaluated (NE)	Edible fruits; potential for overharvesting and habitat loss
*Ficus sycomorus*	Not Evaluated (NE)	Edible fruits; habitat degradation and overgrazing pose threats

Despite these challenges, the study revealed limited active conservation measures. About 90% of respondents reported no deliberate management of WEPs, while only 10% mentioned incidental practices, such as planting along farm boundaries, fences, or within protected pasturelands. Multipurpose species, including *Ziziphus mauritiana*, *Grewia pannosiepala*, and *Delonix elata*, are particularly vulnerable to overuse, underscoring a critical gap in conservation initiatives.

To ensure long-term sustainability, integrated conservation strategies are essential. *In-situ* approaches, including the establishment of protected areas, community-managed forests, and the preservation of sacred groves, can safeguard ecologically and nutritionally significant species, such as *Lannea triphylla*, *Balanites aegyptiaca*, *Moringa stenopetala*, and *Vangueria madagascariensis*. Promoting controlled harvesting practices such as selective plucking and regulated collection can reduce pressure on natural populations while supporting regeneration cycles.

*Ex-situ* strategies, including the establishment of nurseries, seed banks, and botanical gardens, are particularly important for species with restricted distributions or high vulnerability, such as *Boswellia ogadensis*, *Commiphora myrrha*, and *Echidnopsis dammanniana*. Cultivation and propagation of highly nutritious and economically valuable plants, including *Amaranthus dubius*, *Cordeauxia edulis*, and *Tamarindus indica*, offer sustainable alternatives to wild collection and provide additional livelihood opportunities for local communities.

### Jaccard similarity analysis of wild edible plants

The comparison of wild edible plant (WEP) species documented in Korahe Zone with findings from previous ethnobotanical studies across Ethiopia revealed varying levels of similarity (Table 13). The Jaccard Similarity Index (JSI) ranged from 0.046, observed with Sedie (63), to 0.407 with Lowland Ethiopia (58). The relatively high similarity with Lowland Ethiopia likely reflects shared ecological conditions and analogous cultural practices among pastoral and agro-pastoral communities that shape the use of WEPs.

**Table 13:** Jaccard similarity index comparing wild edible plants documented in Korahe Zone with previous ethnobotanical studies in Ethiopia

Study area	Species number (a* or b)	Common species(c)	Jaccard Index	References
Korahe	57*	–	–	Present study
Adola	46	8	0.084	[40]
Arsi	36	7	0.081	[41]
Awi	39	6	0.066	[42]
Berek	34	5	0.058	[17]
Bullen	29	5	0.061	[43]
Burji	46	10	0.107	[44]
Chilga	33	5	0.058	[45]
Delanta	49	6	0.06	[46]
Dibatie	54	7	0.067	[47]
Dire Dawa	22	12	0.179	[48]
East shewa	40	21	0.276	[49]
Gayint	36	5	0.056	[50]
Gibe	74	7	0.056	[51]
Goba	17	10	0.156	[52]
Hararghe	26	14	0.202	[53]
Hula	50	6	0.059	[54]
Kebridehar	30	18	0.261	[26]
Konso	154	10	0.049	[55]
Liben	54	6	0.057	[56]
Lowland Ethiopia	88	42	0.407	[57]
Meinit	66	7	0.060	[58]
Metema	44	6	0.063	[59]
Midakegn	50	8	0.080	[60]
Mieso	41	21	0.272	[24]
Omo	38	14	0.172	[61]
Metema and Quara	51	7	0.069	[30]
Raya	59	6	0.054	[25]
Sedie	33	4	0.046	[62]
Soro	64	7	0.061	[63]
Yalo	16	9	0.140	[64]
Yeki	74	8	0.065	[18]

Moderate similarity values were also recorded with East Shewa (0.276; 58), Mieso (0.272; 25), and Kebridehar (0.261; 27), suggesting considerable overlap in species utilization, possibly influenced by comparable semi-arid environments and intercommunity knowledge exchange. By contrast, much lower similarity values were observed with Sedie (0.046; 63) and Konso (0.049; 56), highlighting the influence of distinct ecological zones, altitudinal variation, and divergent cultural practices on WEP use.

Intermediate similarity was further noted with Dire Dawa (0.179; 49), Hararghe (0.202; 54), and Goba (0.156; 53), which may be explained by geographic proximity and shared cultural traditions, particularly between Somali and Oromo communities, that facilitate overlap in ethnobotanical knowledge and plant use.

### Wild edible plant knowledge among informant groups

Analysis of wild edible plant knowledge across different informant groups revealed significant variations (Table 14). Gender was a strong determinant of ethnobotanical knowledge, with male informants reporting a significantly higher mean number of wild edible plants (4.5 ± 1.7) compared to female informants (2.3 ± 1.3). This difference was statistically significant (*t* = 7.7, *p* < 0.05), indicating greater familiarity with wild edible plants among men.

**Table 14:** Wild edible plant knowledge among informant groups (independent *t* test)

Parameters	Informant groups	*N*	*Mean* ± *SD*	*t value*	*p value*
Gender	Male	68	4.5 ± 1.7	7.7	*P* < *0.05*
	Female	52	2.3 ± 1.3		
Literacy level	Illiterate	76	3.9 ± 1.9	6.2	*P* < *0.05*
	Literate	44	2.1 ± 1.2		
Experience of Informant	Key informant	32	5.5 ± 1.4	9.7	*P* < *0.05*
	General informant	88	2.7 ± 1.3		

Literacy level also influenced knowledge distribution. Illiterate informants demonstrated higher familiarity, reporting an average of 3.9 ± 1.9 species, whereas literate informants mentioned only 2.1 ± 1.2 species. The difference was significant (*t* = 6.2, *p* < 0.05), suggesting that traditional ecological knowledge is retained more strongly among individuals with limited or no formal education.

Experience emerged as the most important factor affecting knowledge levels. Key informants, generally community elders or individuals with recognized ethnobotanical expertise, reported an average of 5.5 ± 1.4 species. In contrast, general informants mentioned only 2.7 ± 1.3 species. This highly significant difference (*t* = 9.7, *p* < 0.05) highlights the central role of key informants in preserving and transmitting ethnobotanical knowledge within Somali communities of the Korahe Zone.

**Fig. 6:**
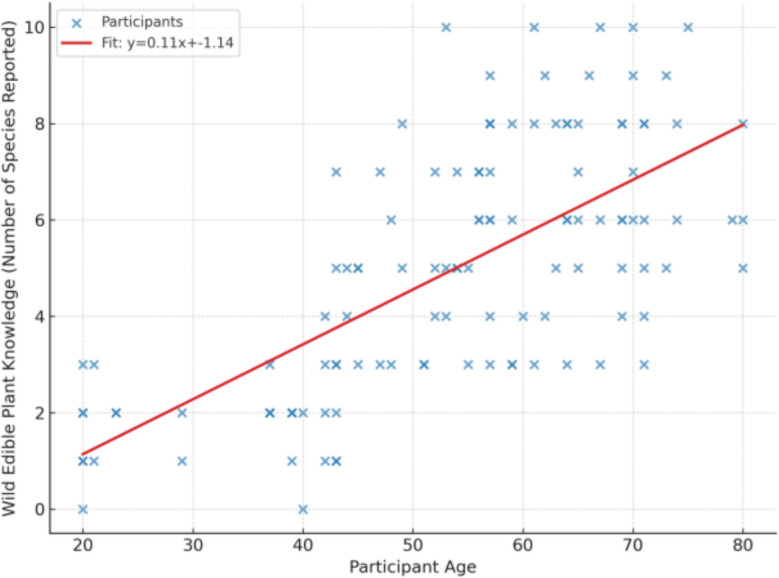
Correlation between age and wild edible plant knowledge of informants

Age was also positively associated with knowledge of wild edible plants. One-way ANOVA indicated significant differences among age groups (*F* = 102.2, *p* < 0.05), with the sum of squares between groups (SS = 657.2) accounting for a larger proportion of total variance than the residual variance within groups (SS = 376.1) (Table 15). Pearson correlation analysis further revealed a strong positive relationship between age and the number of wild edible plants reported (*r* = 0.68, *p* < 0.001) (Fig. 6). The coefficient of determination (*R*^2^ = 0.46) suggests that approximately 46% of the variation in wild edible plant knowledge can be attributed to differences in age, indicating that older informants consistently possess and report greater ethnobotanical knowledge than younger participants.

**Table 15:** Age categories and informant knowledge of wild edible plants (one-way ANOVA)

Source of Variation	Df	SS	MS = SS/Df	F Ratio	*p* value
Between Groups	2	657.2	328.6	102.2	*P* < *0.05*
Residual (within)	117	376.1	3.2		
Total	119	1033.3	331.8		

*Df* = degree of freedom, *SS* = Sum of Squares, *MS* = Mean of Square, Significant codes: 0.05

### Indigenous knowledge transfer and practices of wild edible plants

**Fig. 7:**
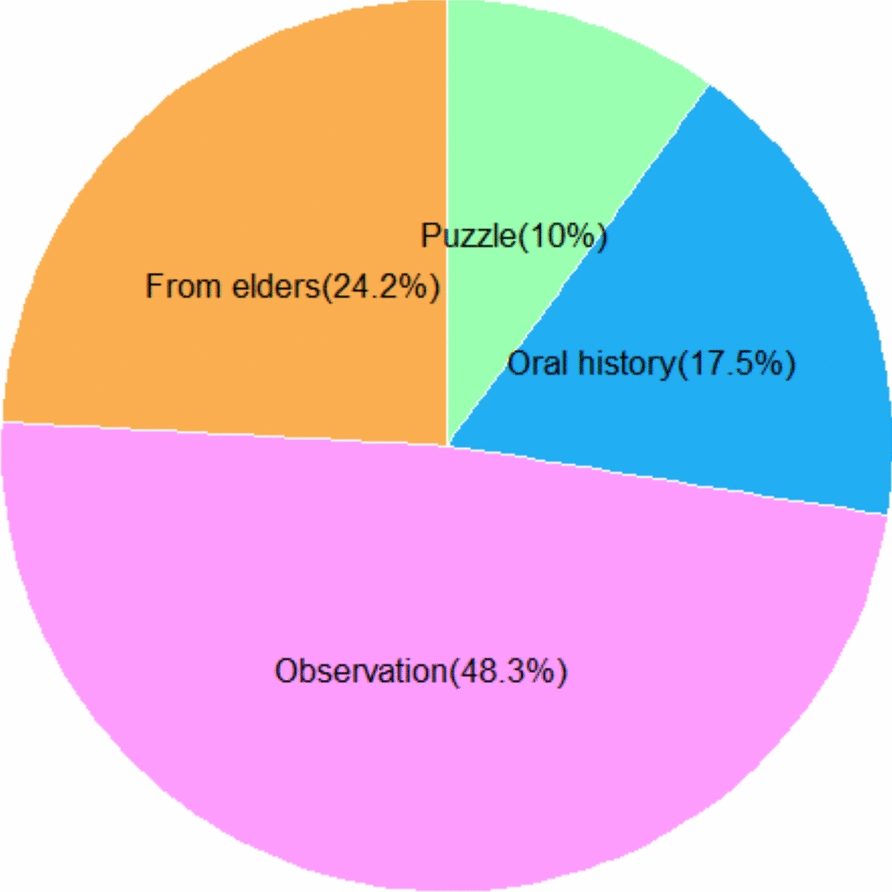
Indigenous knowledge transfer of wild edible plants

The utilization of wild edible plants in Korahe Zone is deeply rooted in traditional knowledge. The use of wild edible plants (WEPs) in Korahe Zone is guided by traditional knowledge systems that govern their identification, harvesting, preparation, and consumption. Among the informants, 58 reported acquiring this knowledge through direct observation, 29 learned discreetly from elders at an advanced age, 21 gained knowledge through oral history, and 12 reported learning through puzzles or stories in the local language, often shared during evening gatherings (Fig. 7).

Indigenous knowledge is primarily transmitted orally across generations, often through family elders, community leaders, and experienced gatherers. This transmission includes teaching the identification of edible species, recognizing which plant parts are safe for consumption, understanding seasonal availability, and distinguishing potentially toxic plants. For example, species such as *Cibirhiza spiculata*, *Iphionopsis rotundifolia*, and *Hydnora abyssinica* require specific processing methods that are learned through observation and practical demonstration.

Traditional practices also encompass sustainable harvesting techniques, including selective plucking, picking, or digging, designed to minimize plant damage and ensure future availability. Communities often follow rules regarding the quantity and timing of collection, particularly for species used during famine, such as *Amaranthus dubius*, *Delonix elata*, and *Cyperus esculentus*, thereby reducing the risk of overexploitation.

Preparation methods reflect sophisticated indigenous knowledge aimed at improving palatability and ensuring safety. Leaves, roots, tubers, fruits, and resins are processed using specific techniques to remove bitterness, toxins, or to enhance taste. For instance, tubers of *Cyperus esculentus* and *Echidnopsis dammanniana* are typically boiled or roasted before consumption, while resins from *Commiphora myrrha* are used sparingly for flavoring or medicinal purposes.

## Discussion

### Diversity of wild edible plants in Korahe Zone

This study documented 57 wild edible plant (WEP) species across 41 genera and 22 families in the Korahe Zone, underscoring the richness of local ethnobotanical knowledge. Fabaceae (10 species, 17.5%), Malvaceae (9 species, 15.8%), and Burseraceae (7 species, 12.3%) were the most represented families, reflecting both ecological abundance in arid and semi-arid landscapes and the reliance of Somali communities on multipurpose species. Comparable dominance of Fabaceae has been reported in southern Ethiopia [65] and other Somali areas [26], highlighting the family’s nutritional, ecological, and cultural significance. Families such as Apocynaceae (6 species, 10.5%) and Rhamnaceae (4 species, 7.0%) showed moderate representation, while others, including Anacardiaceae, Arecaceae, Cyperaceae, and Moraceae, were represented by only one or two species, suggesting more specialized or seasonal use. Similar selective reliance has been observed in arid Kenya [66] and Sudan [67, 68].

The taxonomic distribution in Korahe aligns with findings from other Ethiopian regions, where wild fruits and leaves dominate rural diets. For instance, in Metema and Quara districts of Northwest Ethiopia, 51 WEPs were recorded, with fruits comprising the majority of edible parts [30]. In Konso District, 154 species were documented, with Fabaceae and Moraceae predominant, and knowledge strongly influenced by sociodemographic factors [47]. Such comparisons reinforce the central role of WEPs in Ethiopian food systems while revealing regional differences in family composition and consumption practices.

Comparative evidence from other continents shows how ecological settings and cultural practices shape WEP diversity. In Eastern Bhutan, wild fruits remain important for self-consumption but their use is declining due to crop intensification and dietary change, leading to the erosion of ethnobotanical knowledge [69]. In Gansu–Ningxia–Inner Mongolia, China, 53 WEPs were identified across 24 families, dominated by Asteraceae and Liliaceae, with a broad range of edible parts regularly consumed [70]. In Manipur, India, 86 taxa from 50 families were reported, with Zingiberaceae dominant; notably, 61% of species were marketed and nearly half had medicinal uses, highlighting their contribution to both household nutrition and local economies [71]. In Paraguay’s Atlantic Forest, Mestizo communities utilized 49 species, mostly fruits consumed directly from forests, emphasizing the role of wild resources in forest-dependent diets [72].

Within Ethiopia, the 57 species documented in Korahe are comparable to those reported in Eastern Hararghe (26 species) [53], the lowlands of Ethiopia (88 species) [57], Tach Gayint (36 species) [50], and Midakegn District (50 species) [60]. In contrast, species diversity in Korahe was lower than in Konso (154 species) [55] and Yeki District in Southwest Ethiopia (74 species) [18]. These variations reflect both ecological differences across regions and the adaptive strategies of Somali communities, who have maintained a rich ethnobotanical heritage despite recurring droughts and food insecurity. The findings underscore the need for conservation strategies that prioritize heavily relied-upon families, such as Fabaceae, Malvaceae, and Burseraceae, while also recognizing the value of less represented groups that often provide critical seasonal or famine foods. Integrating community knowledge into conservation and domestication efforts can safeguard biodiversity, strengthen cultural heritage, and enhance the nutritional and economic contributions of WEPs to local food systems.

### Comparative ethnobotanical knowledge across study sites

The analysis using the Botanical Ethnoknowledge Index (BEI) revealed substantial variation in ethnobotanical knowledge among Somali communities in the Korahe Zone. Shilabo exhibited the highest BEI value (0.412), followed by Debe Woyin (0.355), whereas Dumale recorded the lowest value (0.085). This indicates significant inter-community differences in both the depth and distribution of knowledge regarding wild edible plants (WEPs). The mean number of species reported per participant ranged from 3.2 in Dumale to 9.6 in Shilabo, while the total number of species documented mirrored this trend, with 36 species in Shilabo compared to only 12 in Dumale. Similarly, the mean number of citations per species was higher in Shilabo (5.7) than in Dumale (2.1), reflecting not only broader knowledge but also stronger patterns of knowledge sharing within the community.

These patterns suggest that WEP knowledge is jointly shaped by ecological opportunities and sociocultural dynamics. Communities such as Shilabo and Debe Woyin may benefit from greater habitat heterogeneity, facilitating access to a broader diversity of edible taxa, and from stronger traditional knowledge transmission systems, including elder-led teaching and communal foraging practices. By contrast, low BEI values in Dumale may be linked to ecological constraints, reduced species availability, or weakening intergenerational knowledge networks. Comparable patterns have been reported in other parts of Ethiopia: for instance, Eastern Hararghe and the Somali lowlands exhibited pronounced site-specific differences in WEP knowledge, often tied to resource endowment, settlement history, and cultural vitality [26, 53].

Cross-regional comparisons reinforce this interpretation. In Turkana County, Kenya, significant inter-village variation in WEP knowledge has been attributed to differences in habitat diversity, market integration, and intensity of cultural practices [66]. Similar findings from North Kordofan, Sudan, highlight that communities with broader grazing lands and intact customary institutions maintain richer ethnobotanical repertoires than those in more ecologically or socially marginal settings [67, 68]. Beyond Africa, Asian studies echo this dynamic. In Bhutan, ethnobotanical knowledge of wild fruits was found to decline in peri-urban communities compared with remote villages, where traditional gathering and culinary use remain active [69]. Likewise, in rural Tuscany, Italy, intra-regional differences in WEP knowledge were closely tied to the persistence of seasonal food traditions and community cohesion [74].

The uneven distribution of ethnobotanical expertise has direct implications for food security, resilience, and conservation. Communities with higher BEI values, such as Shilabo and Debe Woyin, are better positioned to sustain dietary diversity and buffer food shortages through wild resources while also retaining cultural practices that safeguard biodiversity. Conversely, areas with lower BEI values may be more vulnerable to food insecurity and knowledge erosion. These findings underscore the importance of targeted community-based programs that enhance local ecological knowledge, facilitate intergenerational transmission, and promote sustainable harvesting. Strengthening WEP awareness and practices in less-informed communities will be essential for improving adaptive capacity, supporting sustainable resource use, and preserving the biocultural heritage of Somali communities in Ethiopia’s drylands.

### Lifeforms of wild edible plant species

The documentation of 57 wild edible plant (WEP) species in the Korahe Zone demonstrates substantial ecological and morphological diversity, reflecting the adaptability of Somali communities in utilizing a wide spectrum of plant resources. Lifeform analysis showed that shrubs were the most dominant group (28 species, 49.1%), followed by trees (18 species, 31.6%), herbs (9 species, 15.8%), and climbers (2 species, 3.6%). This distribution underscores the critical role of shrubs and trees in sustaining food security, nutrition, and dietary diversity in Eastern Ethiopia’s drylands.

Shrubs such as *Iphionopsis rotundifolia*, *Cordeauxia edulis*, *Carissa spinarum*, and *Grewia* spp. emerged as the most significant lifeforms. Their prominence reflects their ecological resilience under arid conditions, multipurpose utility, and ability to yield edible fruits, leaves, and seeds with minimal management. Trees, including *Balanites aegyptiaca*, *Moringa stenopetala*, and *Lannea triphylla*, also play central roles by providing fruits, leafy vegetables, fodder, shade, and habitat for biodiversity. Although less abundant, herbs such as *Amaranthus dubius* and *Cyperus esculentus* contribute essential leafy greens and tuberous foods, often serving as supplementary or famine resources. Climbers, though least represented, remain culturally significant in niche ecological settings.

These findings align with other Ethiopian studies, where shrubs and trees consistently dominate WEP composition in dryland and semi-arid regions, such as Somali, Afar, and Eastern Hararghe [26, 53, 64]. Similar lifeform patterns have been documented in the Sudanese Sahel and Kenyan arid zones, where shrubs and trees provide staple famine foods, while herbs and climbers make minor but crucial contributions [66, 68]. Collectively, these studies highlight the resilience and perennial value of woody species in dryland ethnobotanies.

Cross-continental comparisons reveal both convergence and divergence. In Asia, tree and shrub dominance is also observed in arid and semi-arid contexts; for example, in Iran’s Semnan Province, where Rosaceae and Apiaceae shrubs and trees underpin seasonal diets [75], and in Bhutanese mountain communities, where shrubs such as *Berberis* and *Ribes* complement cultivated foods [69]. Conversely, in tropical Asian settings such as Hubei (China), herbs dominate due to humid ecologies that favor herbaceous greens and mushrooms [76, 77].

In Europe, the lifeform balance shifts markedly. A recent survey in rural Tuscany reported a predominance of herbaceous wild vegetables, particularly Asteraceae and Lamiaceae, embedded in local gastronomy [74]. Similarly, Eastern European ethnobotanies emphasize seasonal wild greens and mushrooms over woody taxa, reflecting ecological differences and culinary traditions [5].

American contexts show yet another pattern: studies among Paraguayan mestizo and Guaraní communities documented wild fruits from trees and shrubs as dominant lifeforms, while leafy herbs played only supplementary roles [78]. In Brazil, syntheses also emphasize tree fruits (e.g., *Spondias* and *Eugenia*) as central to diets, shaped by cultural preferences and ecological abundance [79]. These parallels reinforce the role of woody taxa in tropical drylands and forests while highlighting the prominence of herbs in temperate zones.

Taken together, the dominance of shrubs and trees in Korahe reflects broader ecological patterns of dryland ethnobotanies worldwide. Their perennial productivity, multipurpose utility, and resilience against climate stress position them as critical to food security, ecosystem stability, and cultural resilience. Conservation strategies should, therefore, prioritize these lifeforms, particularly nutritionally and culturally significant taxa, such as *Cordeauxia edulis*, *Balanites aegyptiaca*, and *Moringa stenopetala*. Simultaneously, herbs and climbers, though less represented, remain important as supplementary and famine foods and warrant protection. Lifeform-specific management strategies that integrate *in-situ* and *ex-situ* conservation with community-based knowledge transmission will be essential for sustaining biodiversity, enhancing nutrition, and strengthening resilience under Ethiopia’s changing dryland conditions.

### Edible parts and modes of consumption

The present study recorded six primary edible parts of wild edible plants (WEPs) in Korahe Zone: fruits, tubers, roots, leaves, resin, and stems, with fruits predominating (38 species; 66.6%), followed by tubers, roots, and leaves (each 5 species; 8.7%), resin (3 species; 5.2%), and stems (1 species; 1.7%). This strong emphasis on fruits reflects both ecological availability and cultural preference: fruits are often easily accessible, palatable, and typically require little or no processing, which makes them attractive as immediate snacks or supplementary foods. The dominance of fruits in Korahe aligns with multiple Ethiopian and international studies of dryland and semi-arid systems that report fruits as the most commonly used WEP part [26, 53, 80]. Across East Africa, fruits are likewise prominent in WEP portfolios, supporting household diets during lean seasons and providing quick sources of energy and micronutrients [66, 68].

Leaves were the second most commonly used part and are mainly consumed cooked or mixed with other foods, corroborating their recognized role as micronutrient-rich supplements. Species such as *Amaranthus dubius*, *Moringa stenopetala*, and *Corchorus olitorius* documented in Korahe are known sources of vitamins, minerals, and phytochemicals and mirror patterns observed in other Ethiopian settings and the wider African Sahel, where leafy greens are critical for micronutrient intake [81, 82]. Nutrition-focused analyses also emphasize the high protein and micronutrient density of certain wild leaves (e.g., *Moringa*), underscoring their potential for improving maternal and child nutrition [81].

Although fewer species are represented among roots and tubers in Korahe, these parts showed very high Informant Consensus Factor (ICF = 0.95), indicating consistent and shared knowledge about their identification, safe preparation, and use. Tubers and roots such as *Cyperus esculentus* and other locally used taxa are typically cooked to remove bitterness or toxins and serve as important famine or seasonally critical foods across Ethiopia and parts of South Asia [60, 83]. High consensus for these parts is common in regions, where certain tuberous species are relied upon predictably during scarcity, reflecting well-established processing protocols that reduce risk and maximize caloric return.

Resins and stems were used by comparatively few species but had the highest consensus scores (ICF = 0.96–1.00), signifying specialized knowledge and culturally embedded uses often as flavoring agents, chewing gums, or for therapeutic/ritual purposes. This specialization parallels findings from other dryland ethnobotanical studies in East Africa and the Horn, where gums/resins from *Commiphora* and *Boswellia* species serve multiple dietary, medicinal, and economic functions [66, 80].

The Relative Frequency of Citation (RFC) and ICF analyses together emphasize fruits’ central role in local diets (RFC = 0.91), followed by tubers (0.77) and roots (0.67). This hierarchy of fruits as immediate, low-effort food sources and roots/tubers as reserved, processed, or famine resources is widely reported across other Ethiopian districts [48, 53, 60] and in comparative studies from Kenya and Sudan [7, 84]. In South and East Asia, by contrast, life-form and edible-part distributions vary with ecology: temperate and humid areas often have higher proportions of leafy herbs and shoots in WEP lists, while arid regions remain fruit- and tree-dominated [85, 86].

### Collection methods of wild edible plants

The study identified three principal harvesting techniques used by local communities in Korahe Zone: picking, plucking, and digging. These methods are closely tied to the type of plant part collected and the ecological characteristics of the species. Picking emerged as the dominant method (64.9%, 37 species), largely applied to fruits and other above-ground organs that can be gathered without harming the plant. This low-impact practice is consistent with findings from other semi-arid regions of Ethiopia, including the Somali and Afar areas, where hand-harvesting of fruits remains the most common and sustainable method [26, 64]. Similar reliance on hand-picking has been reported in East African countries, such as Kenya and Sudan [7, 66, 67], as well as in arid regions of India and northern China, where selective fruit harvesting ensures continued plant viability while meeting immediate nutritional needs [85, 87]. In contrast, studies from Mediterranean Europe highlight the cultural preference for hand-picking edible greens and fruits, reinforcing the sustainability of this method across both temperate and arid environments [88].

Plucking, accounting for 15.8% of species, was commonly applied to leaves, stems, and resins. This method is especially important for multipurpose species, such as *Amaranthus dubius*, *Moringa stenopetala*, and *Commiphora myrrha*, where careful removal supports both nutritional use and long-term plant survival. Comparable practices have been described in West Africa, where selective leaf plucking contributes to year-round dietary supplementation [82], and in South Asia, where multipurpose plants are sustainably harvested for both food and medicine [83]. In the Americas, particularly in Mexico and Brazil, plucking is similarly practiced for leafy greens and medicinal resins, where traditional knowledge guides selective harvest to avoid plant stress [88, 89].

Digging, reported for 19.3% of species, was used to collect underground organs, such as tubers and roots, including *Cyperus esculentus*, *Echidnopsis dammanniana*, and *Eriosema nutans*. This method is labor-intensive, requiring detailed ecological knowledge of plant morphology, soil conditions, and seasonal cycles. Such practices are critical during times of scarcity, as roots and tubers are energy-dense and often serve as famine foods. However, studies in Ethiopia, India, and the Sahel emphasize that unsustainable digging can threaten plant regeneration and contribute to biodiversity loss if not managed carefully [60, 63, 90]. In Andean South America, similar practices are reported for native tubers, where traditional rotational harvesting techniques are employed to mitigate overexploitation and preserve local agrobiodiversity [91].

### Consumption period of wild edible plants

The study revealed that the majority of wild edible plants (WEPs) in Korahe Zone (41 out of 57 species, 73.2%) are consumed under normal conditions, highlighting their consistent nutritional, cultural, and dietary importance. These regularly consumed species, including *Ozoroa insignis*, *Lannea triphylla*, *Cucumis kelleri*, *Balanites aegyptiaca*, *Moringa stenopetala*, and *Phoenix dactylifera*, serve as staple or supplementary foods, providing essential vitamins, minerals, and energy throughout the year. This pattern aligns with observations from other Ethiopian regions, such as Hararghe, Afar, and the Somali lowlands, where WEPs are routinely incorporated into local diets and culinary practices, reflecting both ecological abundance and cultural preference [26, 48, 53, 57].

Conversely, 15 species (26.8%) were primarily consumed during periods of food scarcity, functioning as emergency or supplemental resources when conventional crops fail. Famine-associated WEPs in Korahe, including *Amaranthus dubius*, *Cordeauxia edulis*, *Cyperus esculentus*, *Delonix elata*, and *Hyphaene reptans*, exhibit high resilience to drought and other environmental stressors, providing critical caloric and micronutrient support. Similar reliance on famine foods has been documented in semi-arid regions of Kenya and Sudan [7, 67], as well as in India and Niger, where communities strategically exploit specific wild species to buffer seasonal and chronic food insecurity [90, 92]. These cross-regional patterns illustrate that the adaptive use of WEPs as either regular or emergency foods is a common strategy in arid and semi-arid ecosystems globally.

Differential consumption patterns underscore the importance of understanding temporal availability and ecological adaptation of WEPs. Species consumed regularly contribute to dietary diversity, support routine nutrition, and reinforce cultural identity, whereas famine-associated species function as safety nets during environmental shocks. In Ethiopia, similar dual roles of WEPs have been reported in Midakegn District and the Somali lowlands, where communities demonstrate sophisticated knowledge of phenology, seasonal abundance, and optimal harvesting periods to maintain food security [57, 60]. In Asia, studies from arid Rajasthan and Tibet show parallel strategies, where local populations rely on drought-tolerant fruits and tubers as fallback foods during crop failure [83, 85]. In the Americas, indigenous communities in Paraguay and the Andean highlands also classify WEPs into regular dietary and emergency-use categories, highlighting the universality of this adaptive approach across continents [73, 78, 91].

Sustainable management of both regularly consumed and famine-associated WEPs is crucial for food security, nutritional health, and cultural preservation. Conservation strategies should prioritize species critical during scarcity, promote rotational and selective harvesting, and integrate indigenous knowledge on phenology and seasonal availability. Documenting consumption periods not only informs local nutritional planning and agro-biodiversity conservation but also provides a framework for climate adaptation strategies in similar arid and semi-arid ecosystems worldwide. Integrating these insights into community-based management ensures that WEPs continue to fulfill their dual role as dietary staples and safety-net foods in the face of environmental variability.

### Seasonal availability of wild edible plants

The seasonal availability of wild edible plants (WEPs) in Korahe Zone reflects the combined influence of ecological conditions, plant phenology, and traditional harvesting practices. In this study, most WEPs were available during spring (September–December; 35.1%), followed by autumn (March–May; 22.8%), summer (June–August; 17.5%), and winter (January–February; 15.8%), with only a small proportion (8.7%) available year-round. This pattern is characteristic of semi-arid agro-pastoral systems, where plant growth, flowering, and fruiting closely track rainfall and temperature cycles [25, 58, 65] Peak availability in spring coincides with post-rainfall regeneration, when fruits, tubers, and leaves are most abundant and palatable, providing critical dietary supplements for local households.

Species such as *Amaranthus dubius*, *Balanites aegyptiaca*, *Boswellia neglecta*, and *Dobera glabra* dominate spring harvests, while summer species, including *Eriosema nutans* and *Cyperus esculentus*, offer essential root- and tuber-based nutrition. Autumn and winter species, such as *Cordeauxia edulis*, *Ficus sycomorus*, *Phoenix dactylifera*, and *Ziziphus mauritiana*, extend the temporal availability of food resources, illustrating the strategic utilization of taxa with staggered phenologies. Notably, species available year-round such as *Moringa stenopetala* and *Lannea triphylla* serve as resilient dietary buffers that stabilize food supply during lean periods.

These observations align with studies from other semi-arid and arid regions of Ethiopia, including Somali, Afar, and Hararghe Zones, where seasonal fluxes of WEPs shape local diets, storage practices, and harvesting schedules [24, 40, 52, 53, 64]. Comparable trends are observed in neighboring East African countries, including Kenya and Sudan, as well as in Niger, where rainfall-dependent WEP availability dictates household nutrition and informs famine mitigation strategies [7, 68]. In South and East Asia, ethnobotanical surveys of arid regions in Rajasthan and the Tibetan Plateau similarly report strong seasonal patterns in fruiting, leaf, and tuber availability, with communities relying on storage, drying, and selective harvesting to buffer seasonal food gaps [83, 91]. In Europe, Mediterranean ethnobotanical studies have documented comparable reliance on seasonally available fruits, herbs, and nuts, highlighting the importance of temporal knowledge for dietary planning and conservation [88]. In the Americas, indigenous communities in Paraguay and the Andes also schedule harvesting according to seasonal peaks, using preservation techniques to maintain access to nutritious WEPs across the year [83, 91].

Relative Frequency of Citation (RFC) analysis in Korahe further underscores the critical role of rainy-season WEPs (RFC = 0.49), reflecting both ecological abundance and cultural preference. These patterns highlight the interplay between environmental conditions, plant phenology, and local knowledge in determining food availability. Understanding these seasonal dynamics is crucial for designing sustainable harvesting regimes, promoting food security, and supporting nutritional planning in arid and semi-arid regions. Conservation and management strategies should prioritize species with narrow seasonal windows, encourage cultivation or enrichment of year-round species, and promote traditional storage and preservation techniques. Integrating seasonal WEP calendars into community education and extension programs can further strengthen resilience against climate variability, drought, and famine.

### Marketability of wild edible plants

The marketability of wild edible plants (WEPs) in Korahe Zone underscores their dual function as nutritional resources and sources of supplemental income for local households. Market surveys identified five actively traded species *Corchorus olitorius*, *Delonix elata*, *Grewia penicillata*, *Ziziphus mauritiana*, and *Tamarindus indica* with women predominantly responsible for their collection and sale. This gendered engagement aligns with patterns observed in other Ethiopian regions, including Hararghe and Southwest Ethiopia, where women manage the marketing of leaves, fruits, and vegetables from wild and semi-wild species [18, 43, 46, 53].

Species such as *Ziziphus mauritiana*, *Balanites aegyptiaca*, and *Cordeauxia edulis* were particularly commercially valuable due to their palatability, high nutritional content, and seasonal abundance. Comparable trends have been reported across semi-arid regions of Kenya, Sudan, and Niger, where the sale of wild fruits and leaves contributes to household resilience, particularly during periods of food scarcity [7, 66, 93]. In Korahe, pricing ranged from 10 to 80 Ethiopian Birr per unit, reflecting both seasonal availability and local demand, with peak sales corresponding to fruiting periods in spring, autumn, and winter.

Species with year-round availability, such as *Moringa stenopetala* and *Lannea triphylla*, offer stable income opportunities and demonstrate the potential for sustainable commercialization. Conversely, species with limited market demand, including *Capparis fascicularis* and *Hyphaene reptans*, are less frequently traded, suggesting that market potential is shaped by cultural preferences, palatability, ease of collection, and storage feasibility.

Globally, the commercialization of WEPs contributes to food security, livelihood diversification, and gender empowerment. In Asia, studies in arid and semi-arid India and Bhutan show that the trade of indigenous fruits and leafy vegetables supplements income and reinforces local dietary diversity [69, 94–96]. In South America, indigenous and mestizo communities in Paraguay and the Andean highlands similarly rely on wild fruit markets for both nutritional and economic benefits, with women playing central roles in collection and commercialization [73, 78, 91]. European Mediterranean studies report that the seasonal sale of wild herbs, fruits, and nuts supports rural livelihoods while promoting biodiversity conservation and cultural gastronomy [88, 97].

These findings highlight the importance of integrating sustainable harvesting practices, value addition, and market access into local WEP management. Promoting the cultivation of highly marketable species in home gardens or agroforestry systems could stabilize supply, reduce pressure on natural habitats, and enhance income generation. Such approaches, implemented globally, demonstrate that strategic commercialization of WEPs can simultaneously advance food security, gender equity, and ecosystem sustainability.

### Adverse effects associated with consumption of wild edible plants

While wild edible plants (WEPs) provide critical nutritional support, food security, and medicinal benefits, their consumption can pose mild to moderate health risks if not appropriately prepared or consumed. In Korahe Zone, informants reported gastrointestinal disturbances associated with certain leafy vegetables and tuberous species, including *Amaranthus dubius*, *Eriosema nutans*, *Alysicarpus rugosus*, *Cyperus esculentus*, and *Cyperus exaltatus*. These effects are primarily attributable to high dietary fiber, oxalates, or other secondary metabolites, reflecting findings from other semi-arid regions of Ethiopia, such as Dire Dawa, Mieso, and Somali lowlands, where similar gastrointestinal discomforts were noted with raw or inadequately processed WEPs [24, 48, 53]. Comparable reports exist from East Africa, including Kenya and Sudan, as well as arid regions of India, where consumption of certain raw tubers and leafy vegetables resulted in temporary digestive upset [7, 37, 68, 71, 83].

Fruits such as *Cordeauxia edulis* and *Delonix elata* were occasionally associated with mild stomach upset when consumed in excess, indicating that portion size and frequency are critical for safe use. Similarly, resinous or latex-containing species, including *Commiphora myrrha*, *Acacia reficiens*, and *Boswellia ogadensis*, were reported to cause localized allergic reactions or irritation. These observations align with studies from Sudan, Benin, Burkina Faso, Niger, and Sahelian regions, where resins and latex from wild plants induced oral or dermal sensitivity [67, 93, 98, 99].

Potential toxicity of certain underground organs, such as *Iphionopsis rotundifolia*, *Sarcophyte sanguinea*, *Cibirhiza spiculata*, and *Echidnopsis dammanniana*, emphasizes the importance of traditional knowledge in preparation methods. Informants consistently reported that proper cooking, roasting, or other processing techniques are necessary to detoxify these species a practice widely documented in Ethiopia, West Africa, and South Asia [99–101].

These findings highlight the indispensable role of indigenous knowledge in ensuring the safe consumption of WEPs. Incorporating educational interventions and community training programs can reinforce appropriate preparation techniques, species-specific precautions, and safe consumption limits. Documenting these traditional practices not only safeguards consumer health but also informs public health policies and sustainable resource management strategies. By integrating local knowledge with modern safety standards, WEPs can continue to provide essential nutrition, dietary diversity, and resilience to food insecurity without compromising safety.

### Preference ranking of wild edible plants

The preference ranking of wild edible plants (WEPs) in Korahe Zone reflects the Somali community’s selective utilization patterns, which are influenced by palatability, nutritional value, availability, and ease of collection. Species such as *Cordeauxia edulis*, *Balanites aegyptiaca*, *Amaranthus dubius*, *Moringa stenopetala*, and *Phoenix dactylifera* emerged as the most preferred, demonstrating their multifunctional roles as staple or supplementary foods, nutrient-dense sources, and, in some cases, income-generating resources. These findings are consistent with studies in other Ethiopian drylands, including Somali and Afar regions, where these species are similarly valued for their protein, carbohydrate, and micronutrient content [64, 102].

Moderately preferred species, including *Ziziphus mauritiana*, *Ficus sycomorus*, *Lannea triphylla*, and *Commiphora myrrha*, illustrate how seasonality, taste preferences, and accessibility shape utilization patterns. Seasonal scarcity or the need for specific processing techniques can reduce the immediate appeal of these species, despite their nutritional and cultural significance. Similar patterns have been documented in Kenya, Sudan, and India, where WEPs with limited availability or complex preparation requirements are less frequently prioritized by local communities [7, 68, 83].

The least preferred species, such as *Cyperus esculentus*, *Echidnopsis dammanniana*, and *Boswellia ogadensis*, are predominantly consumed during famine periods or require labor-intensive processing to mitigate bitterness or toxicity. This aligns with global ethnobotanical trends, where low-preference species are generally reserved as emergency foods or necessitate specialized preparation, as reported in West Africa, South Asia, and parts of South America [96, 99–101].

Understanding these preference patterns is critical for prioritizing conservation and sustainable management. Highly preferred species face greater harvesting pressure and, therefore, require targeted *in-situ* and *ex-situ* conservation strategies. Conversely, lower preference but nutritionally valuable species should be promoted through community awareness, training in processing techniques, and integration into local agroforestry or home garden systems. Such measures can enhance dietary diversity, strengthen food security, and provide continuous nutritional and economic benefits while reducing overexploitation of wild populations.

### Direct matrix ranking of multi-purpose wild edible plants

The Direct Matrix Ranking (DMR) analysis highlights the multifunctionality of wild edible plants (WEPs) in Korahe Zone, emphasizing their integral role in sustaining local livelihoods, culture, and ecological knowledge. Species such as *Balanites aegyptiaca*, *Cordeauxia edulis*, *Commiphora myrrha*, *Boswellia ogadensis*, and *Moringa stenopetala* ranked highest due to their diverse applications, which extend beyond dietary uses to include medicinal purposes, fuel, construction materials, fodder, and cultural practices. These results align with ethnobotanical studies across Ethiopia, particularly in arid and semi-arid regions, where multipurpose WEPs are central to food security, income generation, and traditional medicine [24, 26, 53, 57, 64].

The high cumulative DMR scores underscore both the ecological and socio-economic importance of these species, but also suggest potential vulnerability. Heavy reliance on a limited number of multipurpose WEPs can lead to overharvesting, habitat degradation, and population decline. Similar trends have been observed in Sudan, Kenya, and semi-arid regions of India, where high-ranking multipurpose species experience significant extraction pressures and face conservation challenges [7, 67, 100]. These findings highlight the need to balance utilization with sustainability, particularly for species that support multiple ecosystem services.

Moderately ranked species, including *Amaranthus dubius*, *Ziziphus mauritiana*, *Lannea triphylla*, and *Ficus sycomorus*, are primarily valued for food and have a narrower functional scope. Although these species experience lower extraction pressure, their limited multifunctionality may reduce their prioritization in local management, potentially compromising long-term conservation if they are overlooked. Comparable patterns have been reported in West Africa and South America, where species with restricted functional roles receive less conservation attention despite their nutritional or cultural importance [96, 99, 101].

The DMR findings emphasize the need for integrated conservation and management strategies. Community-based management, controlled harvesting regimes, and cultivation of high-demand species in home gardens or agroforestry systems can reduce pressure on wild populations. In addition, documenting the multifunctional uses of WEPs strengthens the justification for their inclusion in national and regional biodiversity conservation, food security, and livelihood programs. Such an approach ensures that culturally and ecologically valuable species continue to provide nutrition, income, and ecosystem services while maintaining ecological resilience.

### Most popular wild edible plants

The assessment of the most popular wild edible plants (WEPs) in Korahe Zone highlights species integral to local diets, nutrition, and cultural practices. Species such as *Cordeauxia edulis*, *Balanites aegyptiaca*, *Phoenix dactylifera*, *Ziziphus mauritiana*, *Lannea triphylla*, *Ficus sycomorus*, and *Vangueria madagascariensis* were the most cited and widely used, reflecting their palatability, nutritional value, seasonal or year-round availability, and ease of harvest. This pattern mirrors findings from Ethiopia’s Somali and Afar regions, where multipurpose fruits serve as dietary staples and culturally significant resources [24, 26, 48, 49, 53, 64].

Leafy vegetables, including *Amaranthus dubius*, *Corchorus olitorius*, and *Moringa stenopetala*, also ranked highly due to their rich micronutrient content, particularly vitamins and minerals. Similar trends have been observed across semi-arid regions of Africa, including Kenya and Sudan, and in parts of Asia, such as India, where leafy WEPs contribute substantially to nutritional diversity in rural diets [66, 67, 86, 90].

Roots and tubers, such as *Eriosema nutans*, *Alysicarpus rugosus*, *Cyperus esculentus*, and *Echidnopsis dammanniana*, were primarily consumed during famine periods, emphasizing their role as emergency or supplemental foods. This adaptive strategy parallels findings from southern Ethiopia and West Africa, where tuberous WEPs serve as critical safety nets during seasonal or environmental food shortages [49, 99].

Resin-producing species, including *Commiphora myrrha* and *Boswellia ogadensis*, were valued for both nutritional and medicinal purposes. Their multifunctional use underscores the interconnectedness of traditional knowledge, health, and livelihoods. Similar multifunctionality has been documented in arid regions of Africa, the Middle East, and India, where resins serve dietary, therapeutic, and economic roles [103, 104].

Identifying the most popular WEPs provides crucial guidance for conservation and sustainable management strategies. High-utilization species are particularly vulnerable to overharvesting and habitat degradation. Incorporating local preferences into conservation planning, promoting the cultivation of high-demand species, and developing market-based value chains can simultaneously enhance food security and community livelihoods. In addition, documenting their multifunctional uses supports the preservation of indigenous knowledge, ensuring the resilience of local food systems in the face of ecological and socio-economic challenges.

### Newly identified wild edible plants

The identification of nine newly documented wild edible plant (WEP) species in Korahe Zone underscores the dynamic nature of indigenous knowledge and the continuing importance of wild resources for food security in arid and semi-arid regions. Newly reported species including *Cordeauxia edulis*, *Boswellia neglecta*, *Commiphora myrrha*, *Commiphora serrulata*, *Dobera glabra*, *Ziziphus hamur*, *Grewia tenax*, *Carissa spinarum*, and *Hyphaene reptans* were previously unrecorded in the zone but are actively utilized by local communities. These findings expand the documented ethnobotanical range for the Somali Region and align with similar discoveries in other semi-arid regions of Ethiopia, including Afar and Gibe, where previously overlooked WEPs have been recognized as culturally and nutritionally significant [41, 51].

The use of *Cordeauxia edulis* seeds as famine food reflects patterns observed in Kenya and southern Yemen, where this species provides a reliable source of protein and calories during food scarcity [68, 105]. Similarly, *Dobera glabra* and *Ziziphus hamur* exemplify the wider Horn of Africa trend, where perennial woody species serve as critical dietary resources under drought conditions, emphasizing the adaptive value of long-lived plants for sustaining livelihoods in semi-arid ecosystems [7, 49, 98].

The inclusion of resinous species (*Commiphora myrrha*, *C. serrulata*, and *Boswellia neglecta*) highlights the multifunctional nature of WEPs, contributing to nutrition, medicinal applications, and flavoring. Comparable multifunctional uses have been documented in arid regions of Sudan, India, and the Middle East, illustrating the broader global pattern, where resin-producing plants support both dietary and economic needs [90, 103]. These species, particularly *Cordeauxia edulis*, which is considered threatened, underscore the urgency for targeted conservation strategies, including sustainable harvesting, in situ protection, and community-based management.

The discovery of these newly recognized WEPs carries significant implications for food security, biodiversity conservation, and livelihood resilience in Korahe Zone. Incorporating these species into local agroforestry systems, community seed banks, and conservation programs can safeguard them against overexploitation and climate-related pressures. Furthermore, documenting newly identified plants reinforces the importance of preserving indigenous knowledge and highlights opportunities for nutritional, economic, and cultural benefits in semi-arid and drought-prone regions worldwide.

### Implications of using wild edible plants

The 57 documented wild edible plant (WEP) species in Korahe Zone play a crucial role in sustaining food security, particularly amid recurrent droughts and seasonal food shortages. Species such as *Cyperus esculentus*, *Echidnopsis dammanniana*, and *Amaranthus dubius* serve as essential emergency foods, providing energy and critical nutrients when conventional agricultural products are scarce. This aligns with findings from other arid regions of Ethiopia, including the Amhara and Tigray regions, where wild tubers and leafy vegetables function as vital famine foods [25, 45]. Comparable patterns are observed across East Africa, including Kenya and Sudan, as well as in West African Sahelian communities, where WEPs buffer households against food insecurity and diversify diets during resource-scarce periods [66, 68, 99].

Nutritionally, many WEPs contribute significantly to micronutrient intake. Leaves of *Moringa stenopetala* and *Amaranthus dubius* are rich in vitamins A, C, and iron, while fruits from *Balanites aegyptiaca*, *Cordeauxia edulis*, and *Grewia tenax* supply carbohydrates, minerals, and antioxidants. Similar nutritional contributions have been documented in India, Southern Africa, and Latin America, where wild fruits and leafy vegetables enhance diet quality and prevent micronutrient deficiencies [96, 106]. These findings emphasize the global importance of WEPs in improving nutrition, particularly in resource-limited settings.

Several wild edible plants identified in this study contain bioactive compounds with both nutritional and pharmacological properties. For example, *Amaranthus dubius* contains flavonoids and phenolic compounds with antioxidant activity, while *Cordia edulis* has saponins and alkaloids exhibiting antimicrobial effects. *Acacia senegal* provides essential nutrients and contains tannins with antimicrobial activity, and *Ziziphus mauritiana* is consumed as food while traditionally used to manage digestive disorders. However, some species may have adverse effects if consumed in excess; for instance, ingestion of *Calotropis procera* latex can be toxic, and other species may cause gastrointestinal irritation. These dual roles of wild edible plants as both food and medicine reflect the rich ethnobotanical knowledge of local communities and highlight the importance of understanding plant metabolites and safe consumption practices [23, 65, 102].

Beyond their nutritional value, WEPs are deeply embedded in cultural and traditional practices, reinforcing social cohesion and facilitating intergenerational knowledge transfer. Species such as *Tamarindus indica*, *Ficus sycomorus*, and *Commiphora myrrha* serve dual roles as food and cultural symbols in rituals, echoing ethnobotanical observations from North Africa, the Middle East, and Mediterranean Europe [88, 97, 103]. This underscores the broader significance of WEPs as both dietary staples and instruments of cultural identity across diverse regions.

Environmentally, the sustainable harvesting and integration of WEPs into home gardens or agroforestry systems support critical ecosystem services, including soil stabilization, biodiversity conservation, and carbon sequestration. Species such as *Balanites aegyptiaca* and *Lannea triphylla* contribute to ecological resilience, demonstrating the synergy between traditional plant use and environmental sustainability. Comparable observations in Ethiopia, India, and Latin America reveal that multipurpose wild plants can reduce pressure on croplands while maintaining ecological balance and resilience to climate variability [95, 107].

### Conservation and management of wild edible plants

Wild edible plants (WEPs) in Korahe Zone are indispensable for food security, nutrition, and cultural heritage, yet their sustainability is increasingly threatened by both anthropogenic and environmental pressures. Overharvesting of highly valued species, including *Cordeauxia edulis*, *Balanites aegyptiaca*, *Amaranthus dubius*, and *Moringa stenopetala*, has emerged as a major concern. Similar patterns have been documented in other parts of Ethiopia and East Africa, where intensive collection of fruits, leaves, and tubers without adequate regeneration has caused population declines and reduced availability of key WEPs [30, 54, 68]. Comparable pressures are observed in semi-arid regions of West Africa, India, and the Middle East, where overexploitation threatens both wild food resources and ecosystem integrity [90, 99, 100, 103].

Habitat loss due to agricultural expansion, deforestation, and urbanization further endangers species, such as *Lannea triphylla*, *Vangueria madagascariensis*, and *Commiphora myrrha*. Climate change, particularly erratic rainfall and extreme temperatures, negatively affects seasonal fruiting and tuber development in species, such as *Cyperus esculentus*, *Echidnopsis dammanniana*, and *Hydnora abyssinica*, reflecting similar observations in North African and Asian arid regions [10, 76, 77, 103].

Livestock grazing and trampling restrict the regeneration of herbaceous and shrub species, including *Amaranthus dubius*, *Eriosema nutans*, and *Corchorus olitorius*, while invasive plants such as *Parthenium hysterophorus* and *Prosopis juliflora* compete for resources, reducing both the availability and quality of WEPs.

According to IUCN Red List assessments, three species in the zone are threatened: *Boswellia ogadensis* (Critically Endangered), *Cordeauxia edulis*, and *Commiphora cyclophylla* (Vulnerable). Other frequently harvested species remain at risk from unsustainable use despite being categorized as Least Concern or Not Evaluated. Similar conservation challenges are reported in West Africa, India, and the Horn of Africa, highlighting the need for continuous monitoring and reassessment of WEP populations [30, 90, 99, 100, 108].

Current local conservation practices are limited. Approximately 90% of respondents reported no structured strategies, relying only on incidental measures, such as planting along farm boundaries. This gap underscores the need for integrated, community-based, and policy-supported interventions. Effective strategies include *in-situ* conservation through community-managed forests, sacred groves, and protected areas to safeguard ecologically and nutritionally valuable species, such as *Lannea triphylla*, *Balanites aegyptiaca*, *Moringa stenopetala*, and *Vangueria madagascariensis*. Sustainable harvesting practices, including selective plucking, careful digging, and rotational collection, help reduce overexploitation while allowing natural regeneration.

*Ex-situ* conservation strategies, such as nurseries, seed banks, and botanical gardens, are essential for threatened or restricted species, including *Boswellia ogadensis*, *Commiphora myrrha*, and *Echidnopsis dammanniana*. Propagation and cultivation of high-value species (*Amaranthus dubius*, *Cordeauxia edulis*, *Tamarindus indica*) can relieve pressure on wild populations while ensuring reliable food and income sources. In addition, community engagement and awareness programs can educate local populations on sustainable use and integrate WEPs into regional food security and conservation programs, preserving cultural knowledge, maintaining ecological balance, and ensuring long-term availability.

Thus, combining *in-situ* and *ex-situ* conservation with sustainable harvesting, cultivation, and active community participation is essential to protect WEP diversity, enhance resilience against food insecurity, and sustain traditional ethnobotanical knowledge. These approaches align with best practices documented in Ethiopia, Africa, Asia, and globally, highlighting the universal importance of integrating conservation with livelihoods [18, 44, 46, 77, 85, 95].

### Jaccard similarity analysis of wild edible plants

The Jaccard similarity analysis revealed notable variation in wild edible plant (WEP) composition between Korahe Zone and other regions of Ethiopia, reflecting the combined influence of ecological heterogeneity and culturally mediated plant use. The highest similarity (JSI = 0.407) was observed with Lowland Ethiopia [57], likely due to shared environmental conditions, including arid and semi-arid climates, and comparable pastoral and agro-pastoral livelihood systems. These ecological and socio-cultural parallels facilitate the selection and utilization of similar WEP species, underscoring the interplay between environment and tradition in shaping ethnobotanical knowledge.

Moderate similarity values with East Shewa 0.276; [49], Mieso 0.272; [24], and Kebridehar 0.261; [26] indicate partial overlap in species usage, likely attributable to shared flora, migratory pastoral practices, and inter-community knowledge exchange. Similar patterns have been documented in other African drylands, including Kenya, Sudan, and Niger, where pastoral mobility and inter-village interactions influence WEP knowledge and species selection [7, 68, 93].

Conversely, very low similarity values with Sedie 0.046 [62] and Konso 0.049 [55] highlight the effects of distinct ecological zones, altitudinal variation, and cultural differentiation on WEP utilization. Differences in local diets, habitat specificity, and traditional knowledge systems contribute to these low overlaps, consistent with ethnobotanical studies in East and West Africa, as well as in semi-arid regions of India and China, where ecological and cultural divergence drives species-specific knowledge and reduces cross-site similarity [76, 77, 90, 95].

Regions with moderate similarity, including Dire Dawa 0.179 [48], Hararghe 0.202 [53], and Goba 0.156 [52], may reflect partially shared flora and socio-cultural interactions among Somali and Oromo communities, underscoring the role of geographic proximity and cross-cultural knowledge transmission in shaping WEP use patterns. Comparisons with global studies such as ethnobotanical research in Mediterranean Europe, Latin America, and Southeast Asia indicate similar trends, where neighboring or ecologically analogous communities share higher WEP similarity, while isolated or ecologically distinct regions maintain unique assemblages [77, 96, 97].

High-similarity regions suggest opportunities for collaborative knowledge exchange, conservation initiatives, and sustainable management strategies, while low-similarity zones underscore the importance of documenting unique species assemblages to preserve localized ethnobotanical knowledge. Understanding the ecological and cultural drivers of similarity and divergence provides valuable guidance for regional planning, biodiversity conservation, and future ethnobotanical research, particularly in under-studied arid and semi-arid zones both within Ethiopia and globally.

### Wild edible plant knowledge among informant groups

The analysis of wild edible plant (WEP) knowledge among different informant groups in Korahe Zone revealed significant variations influenced by gender, literacy, age, and experience. Male informants reported significantly higher numbers of WEPs (4.5 ± 1.7) compared to female informants (2.3 ± 1.3; *t* = 7.7, *p* < 0.05). This pattern aligns with previous studies in Ethiopian drylands, including Yeki [18], Metema and Quara [30], Hararghe [53], and Lowland Ethiopia [57], where men, often engaged in pastoralism, foraging, and ecological management, exhibited greater familiarity with plants used for food, medicine, and other purposes. Similar gender-related disparities have been documented across Africa and Asia, reflecting culturally mediated division of labor, with men typically responsible for field and forest foraging, while women focus on household-level plant use [76, 96].

Literacy also influenced WEP knowledge distribution. Illiterate participants reported higher familiarity (3.9 ± 1.9 species) than literate participants (2.1 ± 1.2 species; *t* = 6.2, *p* < 0.05), suggesting that formal education may reduce reliance on traditional ecological knowledge (TEK) in favor of modern practices. This trend has been observed in multiple African contexts, including Uganda and Kenya, as well as in rural regions of India and South America, where older, less formally educated community members retain extensive ethnobotanical knowledge, while younger or more formally schooled individuals report fewer species [66, 95, 107, 109].

Experience emerged as a strong predictor of WEP knowledge. Key informants, typically elders or recognized experts, reported an average of 5.5 ± 1.4 species, significantly more than general informants (2.7 ± 1.3; *t* = 9.7, *p* < 0.05). This underscores the pivotal role of elders in maintaining and transmitting ethnobotanical knowledge. Across Africa, Asia, and South America, elder community members are consistently identified as primary custodians of TEK, guiding identification, harvesting, and preparation of wild plants [41, 50, 60, 68, 107].

Age was strongly positively associated with WEP knowledge (*r* = 0.68, *p* < 0.001; *R*^2^ = 0.46), and one-way ANOVA confirmed significant differences among age groups (*F* = 102.2, *p* < 0.05). Older informants consistently reported a greater number of species, highlighting the accumulation of knowledge through decades of observation and practice. This age-related trend mirrors findings in Ethiopian districts such as Mieso and Kebridehar [24, 26] and is consistent with observations in other parts of Africa, Asia, and Latin America, where elders serve as repositories of ecological knowledge essential for sustaining local food security and cultural practices [107, 109].

The gendered, age-specific, and experience-based distribution of WEP knowledge emphasizes the importance of inclusive conservation and education initiatives. Programs should actively engage both men and women and target younger generations to prevent erosion of traditional knowledge. Systematic documentation of elder knowledge is critical for preserving ethnobotanical heritage, particularly as modernization and formal education may reduce reliance on TEK. Integrating TEK into community-based natural resource management can enhance sustainable harvesting, strengthen food security, and support cultural continuity, as demonstrated in multi-regional studies on wild edible plants [49, 106, 110, 111].

### Indigenous knowledge transfer and practices of wild edible plants

The findings demonstrate that the use of wild edible plants (WEPs) in Korahe Zone is underpinned by rich indigenous knowledge (IK) systems that guide plant identification, harvesting, preparation, and consumption. Knowledge transfer occurs predominantly through observation (48.3%), oral instruction from elders (24.2%), oral history (17.5%), and local narratives or puzzles (10.0%), reflecting the multifaceted and culturally embedded nature of traditional ecological knowledge (TEK). Similar patterns have been observed across Ethiopia, particularly in Meinit, Mieso, Hararghe, and Lowland regions, where oral transmission from elders ensures continuity of ethnobotanical knowledge [18, 46, 57–59, 63]. Comparable modes of knowledge transfer are reported in other African countries, including Kenya and Uganda, as well as in Asian and South American contexts, where observation, apprenticeship, and storytelling remain central to TEK acquisition [109, 110, 112].

The reliance on practical demonstration, particularly for species requiring specialized processing, such as *Cibirhiza spiculata*, *Iphionopsis rotundifolia*, and *Hydnora abyssinica*, underscores the importance of hands-on learning. Globally, communities employ experiential learning to navigate risks associated with potentially toxic or bitter plant parts [43, 113–115]. This knowledge ensures safe utilization of WEPs and demonstrates sophisticated indigenous understanding of plant biochemistry, seasonal availability, and ecological interactions.

Sustainable harvesting techniques in Korahe, including selective plucking, picking, and digging, illustrate community-led strategies to maintain WEP populations. Rules governing collection quantity and timing, particularly for famine foods, such as *Amaranthus dubius*, *Delonix elata*, and *Cyperus esculentus*, prevent overexploitation and support regeneration. Comparable practices have been documented in Ethiopia’s Somali and Oromia regions [24, 26, 40, 41, 49, 116], as well as across sub-Saharan Africa and South Asia, emphasizing the role of traditional management in conserving plant resources while sustaining local diets [7, 67, 90, 95, 110].

Preparation methods further reveal intricate indigenous knowledge. Techniques such as boiling, roasting, and selective resin use enhance palatability and reduce toxicity. For instance, tubers of *Cyperus esculentus* and *Echidnopsis dammanniana* require cooking, while resins from *Commiphora myrrha* are sparingly used. Similar ethnobotanical practices are reported in West Africa, India, and Latin America, where cultural processing ensures both food safety and nutritional optimization [77, 96, 100, 115, 116].

Preservation of indigenous knowledge systems is critical for the safe and sustainable use of WEPs, particularly in arid and semi-arid environments, where food security heavily depends on these resources. Integrating IK documentation with formal education can enhance younger generations’ capacity to utilize WEPs safely, bridging the gap between traditional and modern knowledge systems. Sustainable harvesting rules and processing techniques should inform community-based resource management policies to prevent overexploitation and support biodiversity conservation. The study reinforces the need to prioritize elder knowledge custodians in ethnobotanical research and conservation initiatives, ensuring intergenerational transmission of critical food, nutritional, and health resources.

## Limitations of the study

While this study provides a comprehensive documentation of wild edible plants (WEPs) among the Somali communities in Korahe Zone, several limitations should be noted. First, the reliance on purposively selected informants may have introduced bias, potentially over-representing knowledge from key or elder participants, while under-representing younger or less experienced community members. Second, seasonal and temporal constraints limited field observations to specific collection periods, which may have overlooked species available only outside the survey timeframe. Third, quantitative assessments such as the Botanical Ethnoknowledge Index (BEI) and Relative Frequency of Citation (RFC) depend on participant recall, which could result in under- or over-reporting of certain species or uses. In addition, ecological assessments of plant populations and detailed nutritional analyses were beyond the scope of this study, limiting insights into the sustainability and nutrient composition of the WEPs. Future research incorporating longitudinal surveys, ecological monitoring, and biochemical analyses would strengthen understanding of these resources.

## Conclusion

This study documented a rich diversity of 57 wild edible plant (WEP) species in the Korahe Zone, revealing their central role in sustaining local food systems, nutrition, and cultural identity. By compiling a comprehensive inventory, assessing their cultural significance, examining socio-demographic patterns of knowledge, and identifying the threats they face, the findings confirm that WEPs remain essential for both routine and famine-period diets among Somali pastoral and agro-pastoral communities. The predominance of fruit-bearing shrubs and trees, the consistent use of roots and tubers during scarcity, and the high preference for species such as *Cordeauxia edulis*, *Balanites aegyptiaca*, *Amaranthus dubius*, and *Moringa stenopetala* highlight their nutritional, economic, and cultural importance.

The study demonstrates that ethnobotanical knowledge is unevenly distributed, with older adults, men, and key informants possessing significantly higher expertise. This has important implications for the intergenerational transmission of knowledge, especially as formal education, livelihood changes, and environmental pressures continue to reshape community practices. Likewise, the identification of three threatened species and the heavy multipurpose use of several high-value plants underscore growing ecological pressures from overharvesting, grazing, land conversion, and climate variability.

The implications of these findings are multi-layered. First, recognizing WEPs as integral contributors to nutrition and food security rather than marginal famine foods is critical for regional planning and policy. Their incorporation into local food security strategies, school feeding programs, and nutrition initiatives could strengthen resilience during recurrent droughts. Second, the significant market potential of several WEPs suggests opportunities for income diversification, women’s empowerment, and local value-chain development. Third, the documented threats highlight an urgent need for conservation strategies that combine indigenous knowledge with ecological management, including community-based protection, enrichment planting, and *ex-situ* conservation of threatened and high-use species.

Hence, safeguarding WEPs is vital not only for biodiversity conservation but also for sustaining culturally embedded food systems and enhancing resilience in the face of climate change. Integrating these species into agroforestry systems, promoting their sustainable harvest and cultivation, and strengthening community knowledge transmission will be essential for securing their benefits for future generations. In doing so, the findings contribute directly to the achievement of SDGs related to hunger, health, and sustainable land management.

## Supplementary Information

Supplementary material 1

## Data Availability

No datasets were generated or analysed during the current study.

## Abbreviations

ANOVA: Analysis of variance
BEI: Botanical ethnoknowledge index
CSA: Central statistical agency of ethiopia
IK: Indigenous knowledge
IPNI: International plant names Index
JSI: Jaccard similarity index
POWO: Plants of the World Online
WEP: Wild and semi-wild edible plants
