# Supplementary material for: Wild edible plant species and their role in nutrition and health in Korahe Zone, Eastern Ethiopia
Source: Trop Med Health. 2025 Dec 5;53:182. doi: 10.1186/s41182-025-00867-6 (PMC12683791; doi:10.1186/s41182-025-00867-6)
Supplement: Supplementary file 1 — Supplementary material 1 [file 41182_2025_867_MOESM1_ESM.docx]

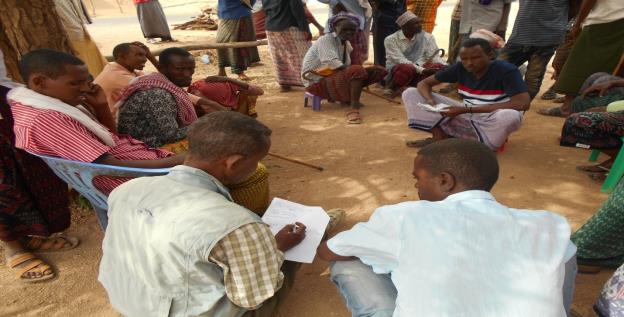


**Figure S1** Interviews with local community members. (Photo credit: Getu Alemayehu, 2023)


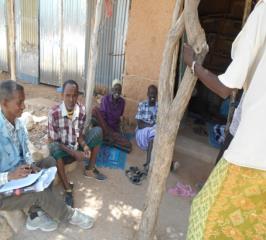

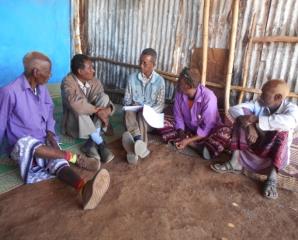


**Figure S2** Photographs illustrating a group discussion with key informants (Photo credit: Getu Alemayehu, 2023)


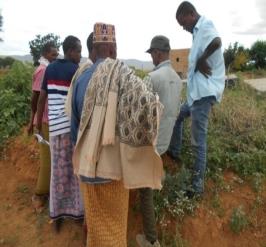

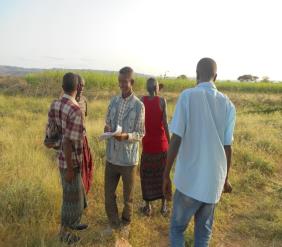


**Figure S3** Guided field walk with a key informant and on-site observations. (Photo credit: Getu Alemayehu, 2023)


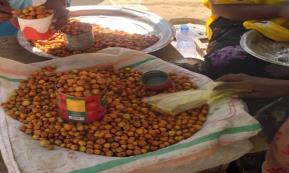

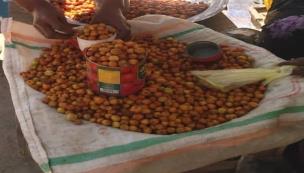


**Figure S4** *Ziziphus mauritiana* Lam. selling at local markets (Photo credit: Getu Alemayehu, 2023)


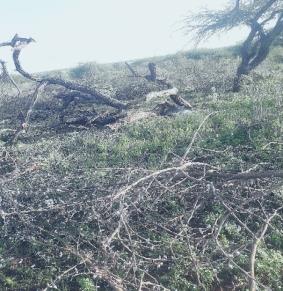

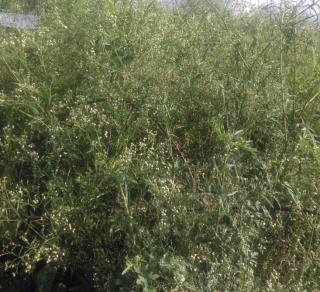


1. Tree cutting for charcoal production b. Invasion by *Parthenium hysterophorus* L.


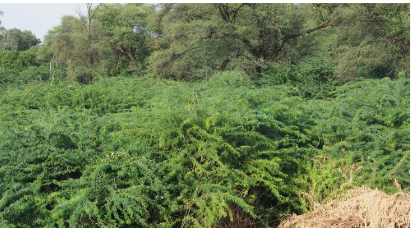


1. Invasion by *Prosopis juliflora* (Sw.) DC.

**Figure S5** Major threats to wild edible plants: (a) tree cutting for charcoal production, (b) invasion by *Parthenium hysterophorus* L., and (c) invasion by *Prosopis juliflora* (Sw.) DC.
